# Eco-Friendly Solvents for Bioactives: Solubilization of Hydroxycinnamic Acids in Glycerol-Derived Ethers

**DOI:** 10.1021/acssuschemeng.5c00269

**Published:** 2025-06-05

**Authors:** Sara Gracia-Barberán, María Lanau, Alejandro Leal-Duaso, Pilar López Ram de Viu, Ana M. Mainar, José A. Mayoral, Elísabet Pires

**Affiliations:** † Instituto de Síntesis Química y Catálisis Homogénea (ISQCH), CSIC-Universidad de Zaragoza, Calle Pedro Cerbuna 12, Zaragoza E-50009, Spain; ‡ Dpto. Química Orgánica, Facultad de Ciencias, Universidad de Zaragoza, c/Pedro Cerbuna, 12, Zaragoza E-50009, Spain; § GATHERS Group, Aragón Institute of Engineering Research (I3A), 16765 Universidad de Zaragoza, c/. Mariano Esquillor s/n, Zaragoza 50018, Spain

**Keywords:** green solvents, solubility, glycerol ethers, hydroxycinnamic acid, ferulic, caffeic, coumaric

## Abstract

Hydroxycinnamic acids, such as coumaric, ferulic, and caffeic acids, stand out for their pharmacological and cosmetic applications due to their bioactive properties. However, their low solubility in water and conventional solvents can be considered a drawback for their effective utilization. This study investigates the solubility of these acids in renewable glycerol-derived ethers, which exhibit good ecotoxicological profiles and tunable physicochemical properties. Experimental solubility data revealed that monoethers and diethers with shorter alkyl chains significantly enhance the solubility of the studied hydroxycinnamic acids. The findings were further corroborated by COSMO-RS modeling, highlighting the importance of both hydrogen-bond donor capacity and polarity-polarizability in solubility enhancement. Hydrotropic effects of glycerol ethers in water were also experimentally demonstrated, indicating their potential in pharmaceutical and industrial formulations. These results underscore the efficacy of glycerol-derived solvents as sustainable alternatives for solubilizing hydroxycinnamic acids, paving the way for greener and more efficient applications.

## Graphical abstract

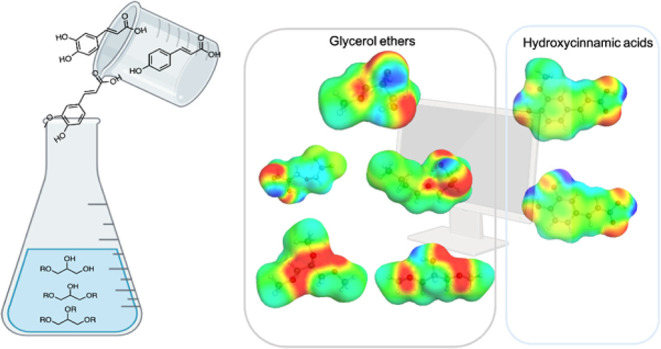

## Introduction

Since the inception of the chemical industry, solvents have been indispensable, serving critical roles in production, extraction, and purification processes. Today, petroleum-derived organic solvents dominate the industry, yet their volatile, toxic, and flammable nature poses significant challenges. Their adverse effects on pollution, air quality, and climate change have spurred the development of sustainable solvents as alternatives to mitigate these environmental and health impacts.^1^

In the pharmaceutical and cosmetic sectors, solvents are even more vital, as they facilitate the solubilization and precise dosing of active ingredients, ensuring their effective delivery into biological systems.^2^ Among the bioactive compounds of interest are phenolic compounds, which are widely found in plants and offer numerous health benefits. Insoluble phenolic compounds, particularly those derived from hydroxycinnamic acids such as ferulic, coumaric, and caffeic acids, are integral components of plant cell membranes and can be obtained from biomass by extraction processes. Hydroxycinnamic acids also exhibit notable antioxidant activity^3–5^ and antimutagenic properties.^6^ These different properties of hydroxycinnamic acids have led to interest in their extraction and solubilization for formulation.

However, extracting or delivering these phenolic compounds is challenging due to their low water solubility and limited stability. Solubility is a critical factor in achieving both the extraction of these hydroxycinnamic acids from biomass and the necessary drug concentration for pharmacological efficacy. Studies by Shakeel et al.^7^ explored the solubility of ferulic acid in conventional solvents such as ethyl acetate, ethanol, isopropanol, butanol, DMSO, and PEG-400, identifying PEG-400 and DMSO as the most effective solvents, with water serving as an antisolvent. Subsequent research^8–13^ confirmed that water is generally ineffective, with methanol and ethanol yielding the highest solubility. Notably, the solubility tends to decrease as the alkyl chain length of alcohols increases.

Recent works have explored the use of neoteric solvents, such as ionic liquids (ILs) and supercritical CO_2_ (scCO_2_),^14^ for solubilizing hydroxycinnamic acids. Imidazolium-based ILs^15,16^ have shown good results, with solubility influenced by the length of the imidazolium substituent and the nature of the anion. For example, ^–^BF_4_ and ^–^OTF-based ILs are particularly effective for coumaric and caffeic acids. In scCO_2_ systems, the addition of cosolvents such as ethanol significantly enhances ferulic acid solubility. Simulations carried out by Petrenko et al.^17^ demonstrated that the increase in the solubility directly depends on the ability of the acid to form hydrogen bonds (HBD) with a cosolvent.

While traditional ILs have proven to provide better solubilization properties than other traditional solvents, certain disadvantages, including their occasionally complex synthesis, toxicity, and nonrenewable origins, limit their use. As a result, biobased ionic liquids, derived from renewable sources, are gaining traction in drug formulation. These ionic liquids are composed of salts from renewable sources and are increasingly relevant in the field of drug formulation.^18^

Additionally, renewable solvents, such as ethyl lactate, γ-valerolactone, and glycerol derivatives, are attracting interest. In particular, glycerol is an interesting biobased solvent derived from the oleochemistry industry of biodiesel production as a byproduct that is frequently used in drug formulations, including oral, parenteral, topical, ophthalmic, and otic preparations. However, its use in extraction processes is often avoided due to its high viscosity and reactivity, leading to a preference for its derivatives, such as ketals (e.g., solketal), esters, and ethers. Glycerol ethers, in particular, are chemically inert and structurally similar to conventional solvents like propylene glycol (PG) and ethylene glycol (EG).^19^ The presence of ether groups in the glycerol moiety allows for tunable properties, such as polarity, hydrophobicity, and viscosity, making them versatile solvents for different applications.

Given these advantages, this study focuses on evaluating the solubility of hydroxycinnamic acids in a selection of glycerol-derived ethers, aiming to provide insights into their potential as sustainable and environmentally friendly solvents.

## Experimental Section

### Materials

Caffeic acid (≥98% purity; CAS No. 331-39-5), coumaric acid (≥98% purity; CAS No. 501-98-4), ferulic acid (99% purity; CAS No. 537-98-4), ethylene glycol (99% purity; CAS No. 107-21-1), 1,3-propylene glycol (98% purity; CAS No. 504-63-2), and ethylene glycol dimethyl ether (97% purity; CAS No. 110-71-4) were purchased from Sigma-Aldrich. Ethylene glycol monomethyl ether (99% purity; CAS No. 109-86-4), methanol, and acetonitrile required for HPLC measurements were purchased from Alfa Aesar, and Milli-Q water was used for these measurements.

### Solvent Synthesis

Mono- and diethers of glycerol have been synthesized following the procedures described elsewhere by Pires et al.^20–22^ Glycerol-derived triethers were obtained from the corresponding diether by methylation with iodomethane.^23^

^1^H and ^13^C NMR spectra of the solvents are gathered in the Supporting Information (Figures S1–S26).

### Solubility Studies

For solubility determination, solutions were prepared by adding 100 mg of each acid to 250 μL of the solvent. Amber vials were used to protect the samples from light. To ensure reliability, all of the experiments were performed in triplicate. The samples were shaken in a biological orbital shaker at 100 rpm for 72 h at 25 °C. Preliminary experiments were performed to determine the equilibrium time, and 72 h was found to be the optimal time. After that time, the samples were microfiltered using a Teflon microfilter (0.4 μm), and appropriate dilutions were made in such a way that the concentration was adequate for subsequent measurements by high-performance liquid chromatography (HPLC).

Before the samples were measured, they must be sonicated to avoid the formation of dimers and ensure the uniformity of the peaks.

### Solubility Determination

The analyses of solutions were carried out by high-performance liquid chromatography (HPLC) using a Waters 2690 Separations chromatograph model in reverse phase with an ultraviolet detector (Waters 2996 Photodiode Array). The column used for the quantification was a C18 Phenomenex Luna (150 nm × 4.6 nm × 5 μm). The volume of injection was set at 10 μL, and the elution was performed at a flow rate of 10 mL/min. The mobile phase was composed of a mixture of methanol:water:0.1% TFA (40:60) in the case of coumaric and caffeic acids and a mixture of acetonitrile:water:0.1% TFA (30:70) in the case of ferulic acid.

Calibration curves were generated using solutions varying in the concentrations of the three studied acids within the HPLC detection range (0–0.006 mg). These solutions were sonicated to ensure uniformity of the peaks and then injected into the HPLC. The linearity of the curves was evaluated by linear regression analysis. Details of the calibrations are gathered in the Supporting Information (Figures S27–S29).

### COSMO-RS Studies

For the application of the COSMO-RS model, the input files for each molecule under study were preoptimized using Gaussian through the HERMES-I3A computing cluster to reduce calculation time. DGA1-DFT with the BP86 functional and the Ahlrichs-TZVP basis set was used.

The optimized molecules were introduced into the COSMOconf tool, where their conformational analysis was carried out. Finally, TmoleX 4.6.0-Turbomole was used to perform geometrical DFT/COSMO optimization. The optimization was carried out for all of the conformers whose energy differed by less than 9 kJ from the conformer with the lowest energy.

All of the optimized molecules were introduced into the COSMOtherm program, where the COSMO-RS method was applied. The sigma profiles and potentials, as well as the estimated solubilities, were obtained.

Cartesian coordinates of the most stable conformer of the molecules are gathered in the Supporting Information.

## Results and Discussion

In this study, three hydroxycinnamic acids with different substitution patterns were selected, namely, coumaric, ferulic, and caffeic acids (Figure 1).

**Figure 1:**
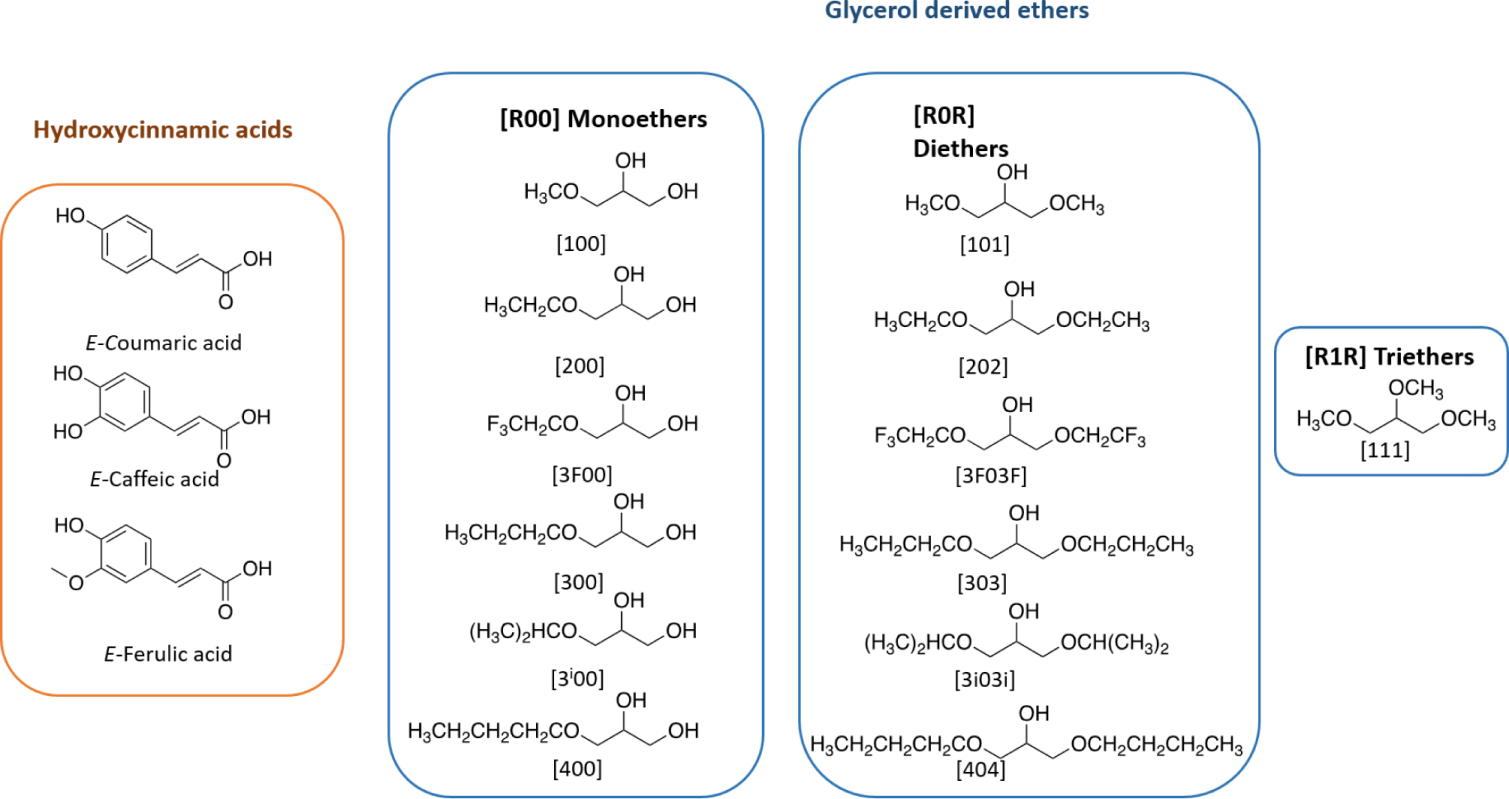
Chemical structures of hydroxycinnamic acids and glycerol-derived ethers used in this work.

As mentioned in the Introduction, glycerol-derived solvents can be considered green solvents due to their renewable origin, easy availability,^20,22^ and good eco-toxicological profile,^24–26^ thus making them excellent candidates to conventional glycols substitution. In the present study, mono- (**R00**), di- (**R0R**), and triethers (**R1R**) have been used as solvents for the measurement of solubility of the selected hydroxycinnamic acids (Figure 1). The selection of glyceryl ethers with different lengths of the **R** substituents, both linear and branched chains, including fluorinated chains, composes an interesting set of solvents with different physical–chemical properties to carry out this study.

The use of these solvents also allows either the extraction of the selected acids and their recovery by precipitation with water from the saturated solutions or their use as a solubilization medium for possible formulation. Viscosities of the selected solvents ranged from 0.7 to 42 cP at 298 K.^20^ Compared to glycerol (1200 cP) and other solvents used in extraction processes, such as PEG-400 (89 cP), this would not prevent the use of glycerol ethers in both extraction and dissolution processes.

For the sake of clarity, a notation was used for glycerol-derived ethers. Thus, **R00** indicates a glycerol monoether, and **R0R** indicates a diether. **R** = 1, 2, 3, 3i, etc. indicates the number of carbon atoms in the alkyl chain of the substituent, i stands for *iso* substituents, and 3F stands for the 2,2,2-trifluoroethyl substituent. All the solvent structures and notations used in this work are gathered in Figure 1.

The solubility determination was conducted in accordance with an optimized protocol, whereby saturated solutions of each hydroxycinnamic acid were stirred in an orbital shaker at a constant temperature (298 K) for 72 h. Following this period, the solution was checked for saturation and filtered. The filtered solution was then immediately diluted for hydroxycinnamic acid quantification by HPLC.

### Solubilities in Glycerol-Derived Monoethers: Influence of the Nature of the Alkyl Chain

The study began with the determination of the solubility of ferulic, caffeic, and coumaric acids in glycerol-derived monoethers **R00**. For the sake of comparison, solubility was also determined in ethylene glycol (**EG**) and 1,3-propylene glycol (**PG**). Glycerol was excluded from this study due to its high viscosity, which hampers reliable solubility values following the protocol used in this work. The solubility values, expressed in milligrams per milliliter, are presented in Figure 2.

**Figure 2:**
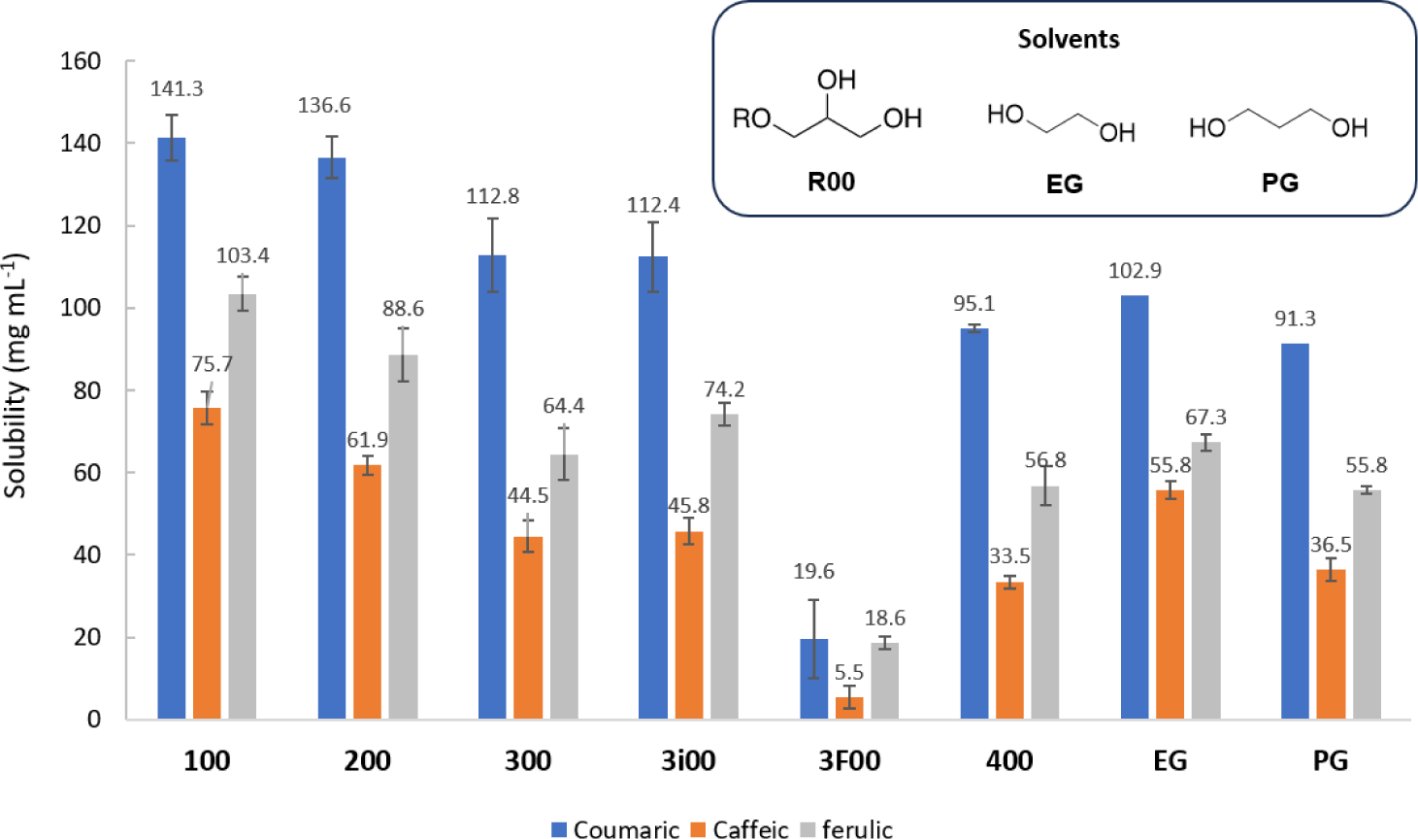
Solubility (mg mL^–1^) of coumaric, caffeic, and ferulic acids in glycerol-derived monoethers, EG, and PG.

First, it can be observed that coumaric acid is the most soluble acid, and caffeic acid is the least soluble compound among the selected hydroxycinnamic acids. This trend is in good agreement with the one observed with conventional solvents and can be directly related to the number of hydroxyl groups in the molecule.^11,12^ Thus, a higher number of hydroxyl groups seems to limit the solubility of the acid, probably due to intermolecular hydrogen bonds.

When solubility values in **R00** glycerol-derived monoethers are compared, it can be evidenced that an increase in the length of the alkyl chain provides a decrease in the solubility in all cases. Almost no differences are observed when using branched substituents (see **300** and **3i00** values), while the presence of fluorinated atoms in the substituent dramatically decreases the solubility (see **200** and **3F00** cases).

In order to explain this behavior, the experimental solubility data were plotted against physicochemical parameters related to different aspects of the polarity of the solvents (Figure 3) such as the log P and Kamlet–Taft parameters, in which α represents the hydrogen bond-donating ability, β represents the hydrogen bond-accepting ability, and π* represents the dipolarity–polarizability of the solvents.^27,28^

**Figure 3:**
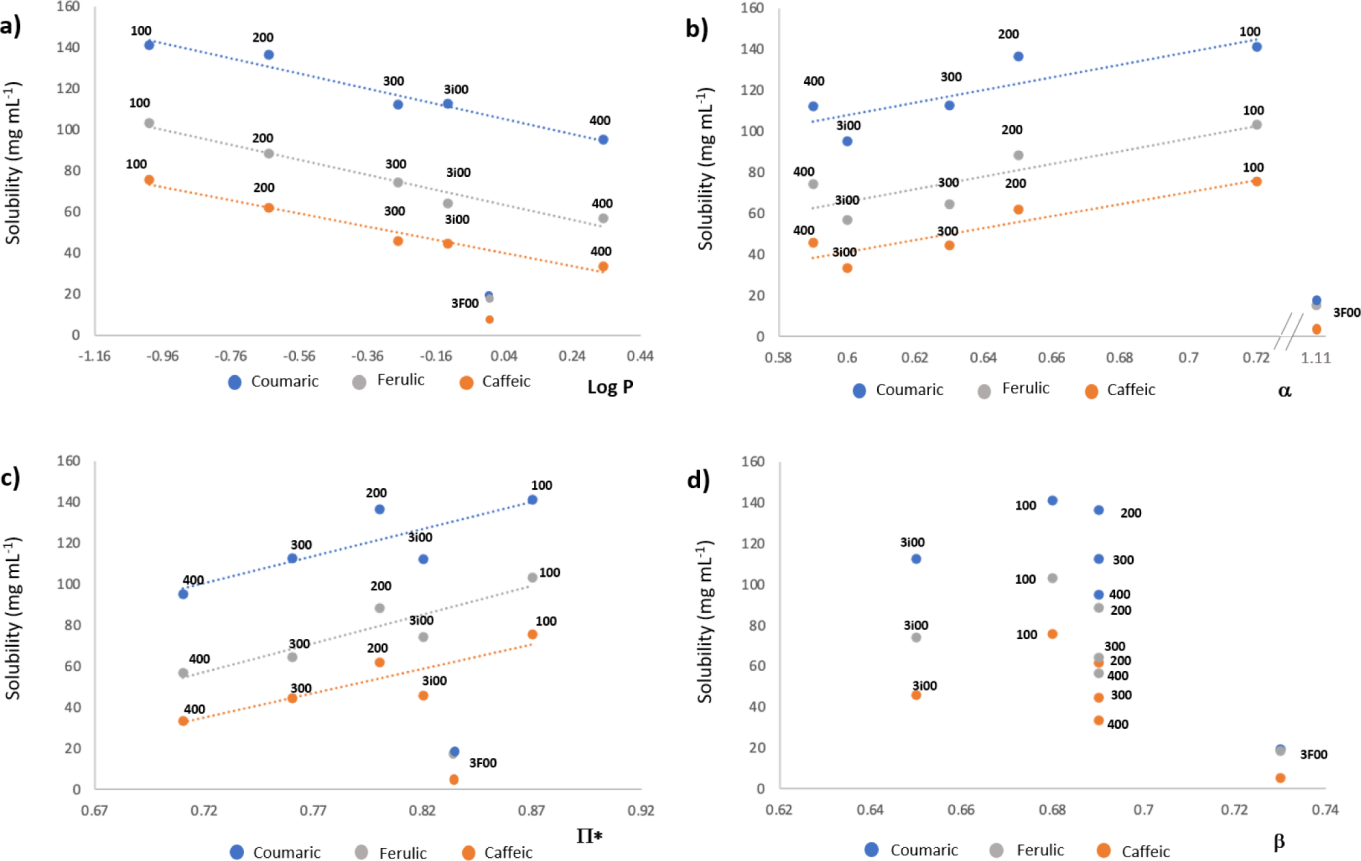
Solubility (mg mL^–‑1^) of coumaric, caffeic, and ferulic acids in glycerol-derived monoethers vs log P (a), α (b), π* (c), and β (d) solvent parameters.

For the three studied solutes, good correlations are observed between log P of the **R00** solvent and the solubility values, except for the fluorinated solvent **3F00** (Figure 3a). Thus, a linear decrease in solubility is observed with increasing solvent hydrophobicity. When other polarity parameters are considered, such as α or π*, a certain correlation is observed, except again for **3F00** (Figure 3b,c); however, it should be noted that no correlation with the β parameter was found in any case (Figure 3d).

So, in the case of monoethers, both the hydrogen bond donor (HBD) capacity and the dipolarity–polarizability seem to be driving forces for the hydroxycinnamic acid dissolution, while the hydrogen bond acceptor (HBA) capacity does not seem to be a determining parameter in this case.

In the case of the fluorinated solvent **3F00**, it should be noted that it presents very particular and different properties from the rest of the monoethers studied. Thus, **3F00** exhibits high polarity and high HBD and HBA capacity, but at the same time, high hydrophobicity. This combination of properties does not seem to be suitable for the solubilization of ferulic, coumaric, and caffeic acids.

In an attempt to corroborate these conclusions with experimental evidence, and based on the studies of Coutinho et al.,^29^ ^1^H NMR studies of the saturated solutions of each of the hydroxycinnamic acids in **100** and **400** were performed using *d*_6_-DMSO. In our case, no displacement of any of the solvent signals was observed, which did not allow us to demonstrate the interactions between the solute and solvent under these analysis conditions (Figures S30–S35).

For the sake of comparison, two nonrenewable glycols with structural similarity to our glycerol solvents were included in this study. In this case, the solubilities of the selected hydroxycinnamic acids were measured in ethylene glycol (EG) and 1,3-propylene glycol (PG). As can be observed in Figure 2, slightly lower solubilities are achieved using these diols compared to our glycerol derivatives; thus, the presence of an additional oxygen in the molecule seems to favor the dissolution of these hydroxycinnamic acids.

### Solubility in Glycerol-Derived Diethers and Triether 111: Influence of the Number and Nature of Substituents

In order to ascertain the contribution of the hydroxy groups present in glycerol-derived ethers to the solubility of the selected hydroxycinnamic acids, the solubility of these solutes was evaluated in the selection of symmetric diethers, which are analogous to the monoethers that were previously tested. Again, solvents with linear and branched alkyl substituents and with fluorinated chains were studied. Solubility values are gathered in Figure 4.

**Figure 4:**
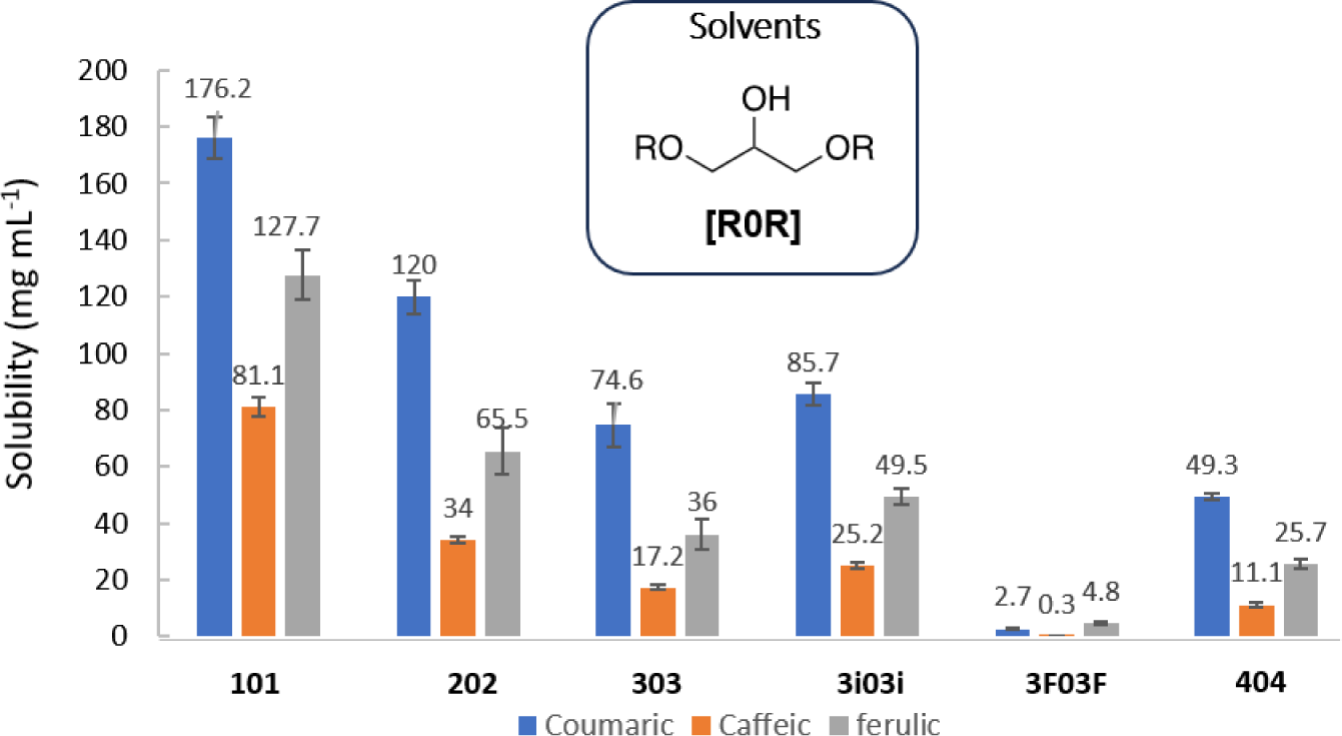
Solubility (mg mL^–1^) of coumaric, caffeic, and ferulic acids in glycerol-derived diethers.

As in the case of glycerol monoethers, an increase in the number of carbon atoms in the R substituent of the diether provokes a decrease in solubility for all of the studied hydroxycinnamic acids.

Again, the presence of fluorinated atoms in the solvent molecule produces a great decrease in solubility (e.g., 122 mg mL^–1^ in **202** vs 2 mg mL^–1^ in **3F03F** for coumaric acid), and in that case, branched chains provide a slight increase in solubility (e.g., 74.6 mg mL^–1^ in **303** vs 85.7 mg mL^–1^ in **3i03i** for coumaric acid) (Figure 4).

When correlating the experimental solubility values in glycerol-derived diethers with the solvent parameters log P, α, β, and π* solvent parameters, some trends can also be observed (Figure 5). Again, in all the cases, the behavior of 3F03F solvent deviates from the behavior of the other solvents.

**Figure 5:**
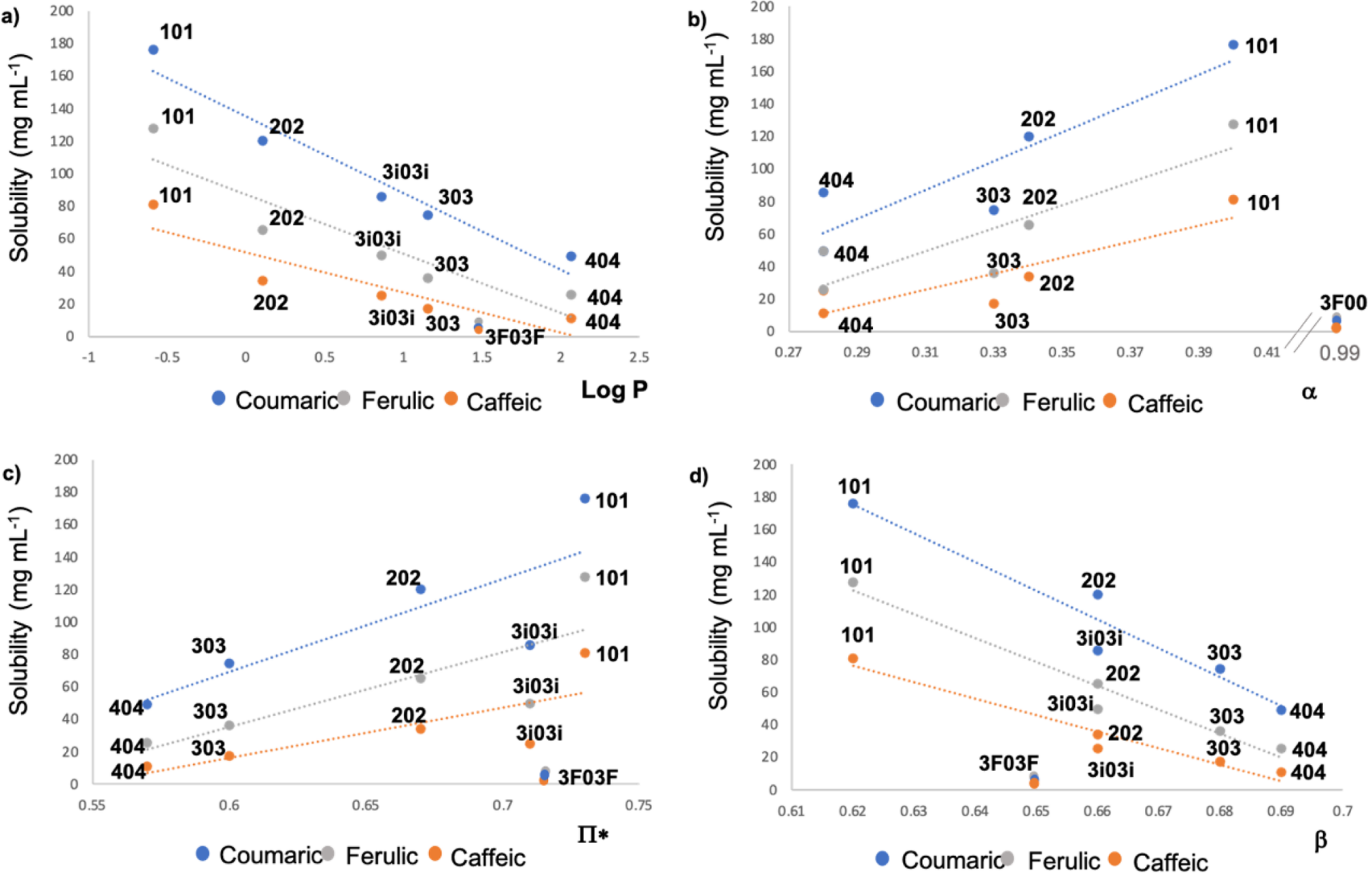
Solubility (mg mL^–1^) of coumaric, caffeic, and ferulic acids in glycerol-derived diethers vs log *P* (a), α (b), π* (c), and (d) β solvent parameters.

In the case of diethers, excellent correlations are observed with HBA capacity (β parameter) and again with log P, although α and β* also show a quasilinear trend. Thus, an increase in hydrophobicity or HBA capacity seems to be detrimental to hydroxycinnamic acid solubility, while an increase in α and π* increased solubility values. Once more, the fluorinated solvent **3F03F** is an exception on account of its distinctive properties.

When comparing solubility values provided by monoethers and diethers, a general decrease in solubility is observed with the increase of both the alkyl chain length and the number of substituents, except when using **101** diether, where an opposite trend is noticed. Two effects can contribute to the decrease in solubility in the case of diethers (except for **101**): first, there is only one hydroxyl group available for establishing solute–solvent interactions, and second, the presence of bulkier substituents contributes to hinder that interaction.

In order to evaluate the necessity of the presence of an OH group in the solvent molecule, solubilities in **111** triether were also determined. The results demonstrated a notable decline in solubility for all of the studied acids. This finding underscores the crucial role of the OH group in solvent molecules in facilitating interactions with the studied solutes and enhancing the solubilization of these compounds.

The solubility in glycol analogues, such as ethylene glycol monomethyl ether (EGMME) and ethylene glycol dimethyl ether (EGDME), was also measured (Figure 6).

**Figure 6:**
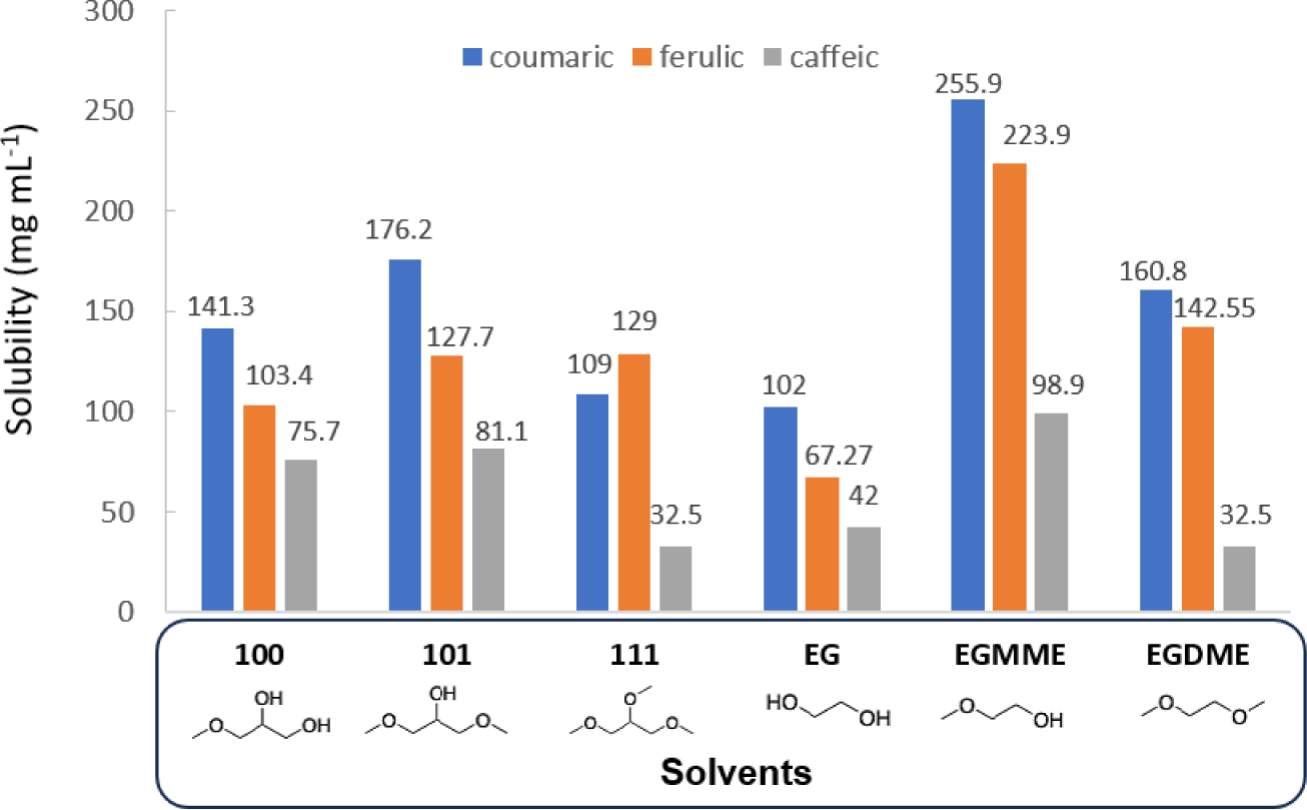
Comparison of solubility (mg mL^–1^) of coumaric, caffeic, and ferulic acids in several methyl ethers.

In that case, EGMME, a nonrenewable glycol, provides the best solubility values, with an increase of 45% for coumaric acid, 75% for ferulic acid, but a lower one (21%) in the case of caffeic acid with respect to **101**.

Finally, in order to compare our best results with those previously obtained in the literature, the solubility values were expressed both in g L^–1^ and in molar fractions and are summarized in Table 1. In this case, only ferulic acid was taken into account, as there are no comparable data in the literature for coumaric and caffeic acids. As can be seen, the best solvent in this case was a nonrenewable one, PEG-**400**. Moreover, of all the renewable solvents studied, glycerol ethers gave the best results.

**Table 1:** Solubility Values of Ferulic Acid Expressed in g L^–1^ and Molar Fraction in a Selection of Glycerol-Derived Ethers, Traditional Solvents, and Renewable Ones

**Solvent**	**Ferulic acid**		**Reference**
	**Concentration(g L**^**–1**^)	**Molar fraction** **(** *X* **)**	
**100**	103.4	0.0543	this work
**101**	127.7	0.0802	this work
**111**	129	0.088	this work
**EGMME**	223.9	0.101	this work
**DMSO**		0.0526	^7^
**PEG-400**		0.154	^7^
**γ-valerolactone**	111		^30^
**Propane-1,2-diol**	81.2		^30^

### Theoretical Study of the Solubilities

COSMO-RS studies were carried out in order to deeply understand the driving forces of the solubilization of coumaric, ferulic, and caffeic acids in the glycerol-derived solvents presented in this study, and also to determine the predictive capacity of COSMO-RS in that case. For this study, solvents **100**, **101**, **111**, **400**, and **3F00** were selected in order to examine the influence of the number of substituents, the length of the alkyl chain, and the presence of fluorine.

First, the conformational study for the selection of the solvents was performed by using COSMOconf. Conformers differing by a maximum of 9 kJ were considered, as recommended by Klamt et al. As stated by them,^31^ this conformational study is crucial in the case of molecules that are able to establish internal hydrogen bonds as in the case of glycerol-derived ethers, because the computed polarization charge densities σ estimated using COSMO-RS strongly depend on the correct representation of these hydrogen bonds. Thus, 12 conformers were considered for **100**, 37 for **400**, 23 for **3F00**, 7 for **101**, and 7 for **111**.

After selecting the more stable conformers in each case, the optimization of the molecular geometry was carried out using TmoleX 4.6.0-Turbomole, and finally, all of them were used in the COSMOTherm calculations to obtain the σ-profiles.

The σ*-*profile provides information about the interaction between the species involved. So that the σ-profile describes the affinity of the molecule for a molecular surface polarity. Thus, positive polarities are represented in the negative part of the σ-profile and vice versa. The σ-profile can be divided into three regions: the hydrogen bond donor (HBD) region (σ ≤ −0.84 e nm^–2^), the nonpolar region, and the hydrogen bond acceptor (HBA) region (σ > 0.84 e nm^–2^). The width of the peak in the central region also conveys information about the molecule polarity; thus, narrow peaks indicate low polarities, while broad peaks indicate high polarities.^32^

When the σ-profile for the glycerol-derived solvents used in this study is observed (Figure 7a), all of the ethers have similar HBA capacity. Looking at the HBD region (σ ≤ −0.84 e nm^–2^), we can observe that monoethers present higher HBD capability (**3F00** ≈ **100** > **400**) followed by diether **101**, and obviously triether **111** showed no HBD capacity. All the solvents present broad peaks in the central region, with this peak being broader for molecules with more or larger substituents (**111** and **400**). In this region, the σ-profile of **3F00** showed a different shape with two peaks, indicating different polarity on the molecule surface due to the presence of fluorine.

**Figure 7:**
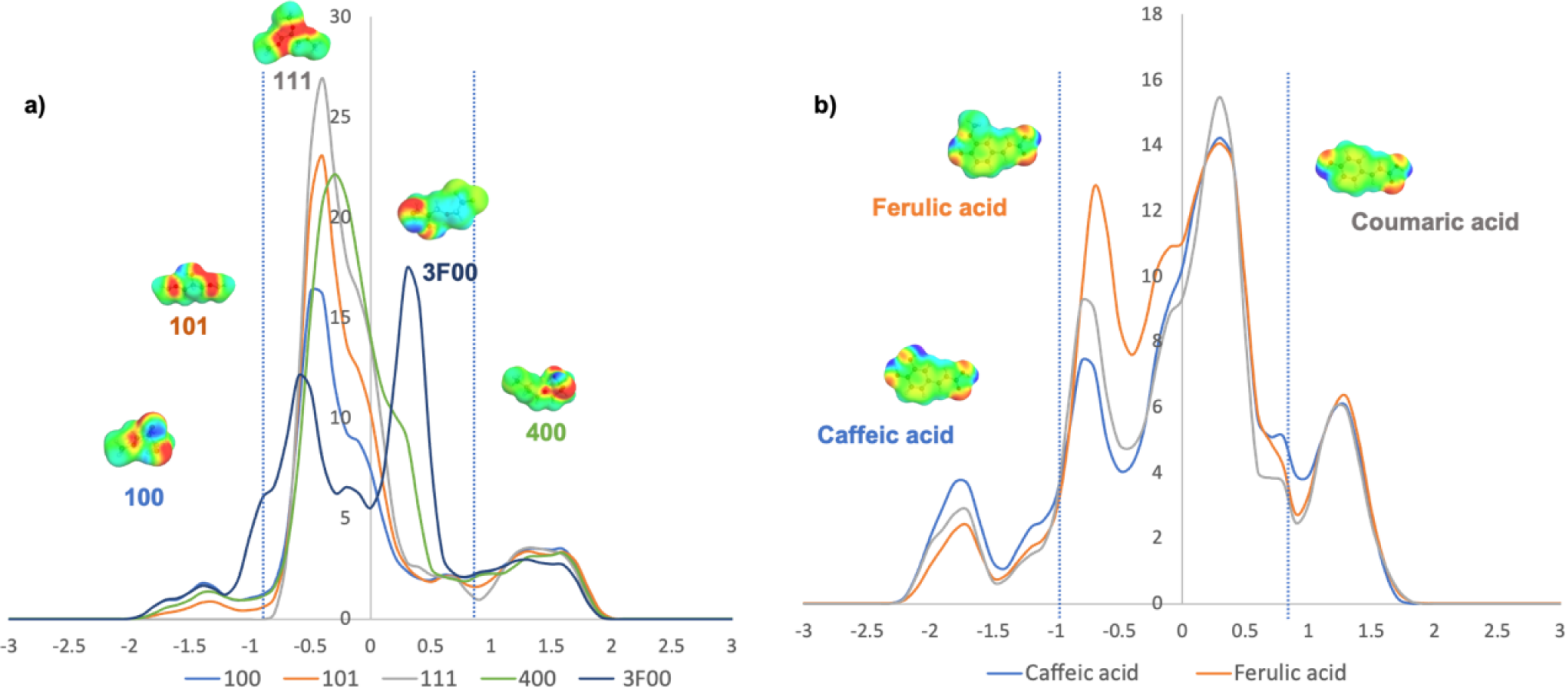
σ-Profile for the glycerol derived solvents (a) and hydroxycinnamic acids (b) used in this study (the vertical dashed lines indicate the threshold for hydrogen bond interaction energy and the COSMO-RS cavities of the most stable conformer).

The main objective of this study was to gain fundamental knowledge of the dominant interaction parameters governing hydroxycinnamic acid solubility in glycerol-derived ethers and the structural features underlying these interactions.

Thus, the conformational study, the geometry optimization, and the σ-profile estimation were also carried out for coumaric, ferulic, and caffeic acids using the same conditions as those used for glycerol ethers.

Figure 7b presents the σ-profile of the hydroxycinnamic acids used in this study. Upon closely examining the HBD region (σ ≤ –0.84 e nm^–2^), COSMO allows prediction that caffeic acid has higher HBD capacity, followed by coumaric and ferulic acids. In the region of HBA (σ > 0.84 e nm^–2^), all three acids showed the same ability to accept hydrogen bonds. Broad peaks are observed in the central part of the profile, thus indicating different polarity regions in the molecules.

Using the information provided by the sigma profiles, and assuming that the driving force for the solubilization of these hydroxycinnamic acids in glycerol-derived ethers is the hydrogen bond formation, it would be expected that caffeic acid would be more soluble in our solvents, as caffeic acid is the acid with the highest HBD capacity and due to the similar HBA capacity of the glycerol ethers. Conversely, if the HBD capacity of the solvent is considered, higher solubilities in monoethers **100** and **3F00** should be observed. That hypothesis is not consistent with the experimental observations. As discussed in the previous section, in the case of monoethers, both HBD capacity and polarity-polarizability are the parameters that better correlate with the solubility values, and in the case of diethers, all the parameters seem to influence the solubility. Thus, the whole σ*-*profile should be taken into consideration in order to predict the solubility of hydroxycinnamic acids in these solvents.

So, in order to know whether COSMO-RS is able to predict, with the lowest computational cost, which would be the best solvent in our case, a noniterative screening method was applied to estimate the solubilities.

As observed (Figure 8a) in all cases, diether **101** is predicted as the best solvent for these hydroxycinnamic acids. The length of the substituent chain and, specially, the presence of fluorine in the solvent molecule reduce the calculated solubility values, as also observed in experimental solubility data.

**Figure 8:**
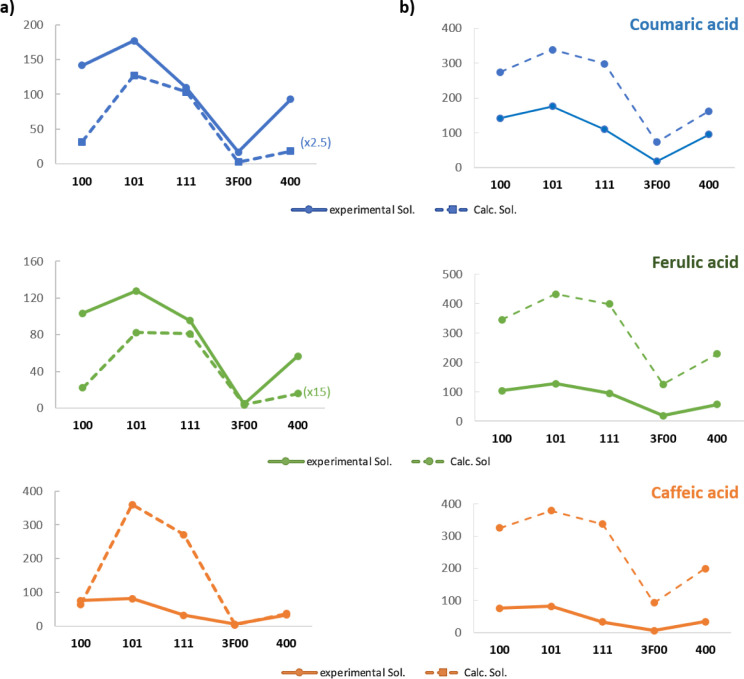
Comparison of the COSMO-RS solubility predictions and experimental data. (a) Relative screening method and (b) SLE method.

However, calculated data showed that the nonrelative screening with COSMO-RS underestimated the solubility of coumaric and ferulic acids for all the solvents, while the prediction was quite accurate for the solubility of caffeic acid in glycerol-derived monoethers. However, it was greatly overestimated in the case of **101** diether and **111** triether (Tables S13–S15). For better comparison, the experimental and calculated data were plotted graphically (Figure 8a).

For a more accurate prediction, absolute solubility values were calculated using SLE (solid-liquid equilibrium) settings (Figure 8b). The same trends as before are observed, but in that case, no correction factors are needed in order to compare with the experimental data for coumaric and ferulic acids. In all the cases, solubilities were overestimated by using SLE (Tables S13–S15).

Despite the existing differences between experimental and theoretical solubility values, the COSMO-RS solvation model, together with ab initio calculations, is capable of predicting trends, which makes this computational tool very powerful as a screening method for the design of new solvents.

### Hydrotropy Studies

In previous works, we demonstrated the hydrotropic ability of glycerol ethers for the solubilization of gallic and syringic acids.^33^ In that case, the results showed that the hydrophobicity of the hydrotrope plays a major role, being dominant in the dilute region.

In order to confirm this behavior also with hydroxycinnamic acids, we carried out a study on the solubilization of coumaric, ferulic, and caffeic acids in water/glycerol ether mixtures. We selected three glycerol-derived ethers, namely, **100** and **400** monoethers and **101** diether, in order to analyze the influence of the number of substituents and the length of the alkyl chain on the hydrotropic ability of the solvents.

The solubility of coumaric, ferulic, and caffeic acids in aqueous solutions of **100**, **101**, and **400** glycerol ethers was determined in the entire molar fraction range at 298.2 K. Solubility values from this hydrotropic study are gathered in the Supporting Information.

Figure 9 presents the solubility data, where *S* represents the solubility (mg mL^–1^) of each studied acid in the water/glycerol ether mixture, *S*_0_ is the value of the solubility (mg mL^–1^) in pure water, and *X* is the molar fraction of glycerol ether in the solvent mixture.

**Figure 9:**
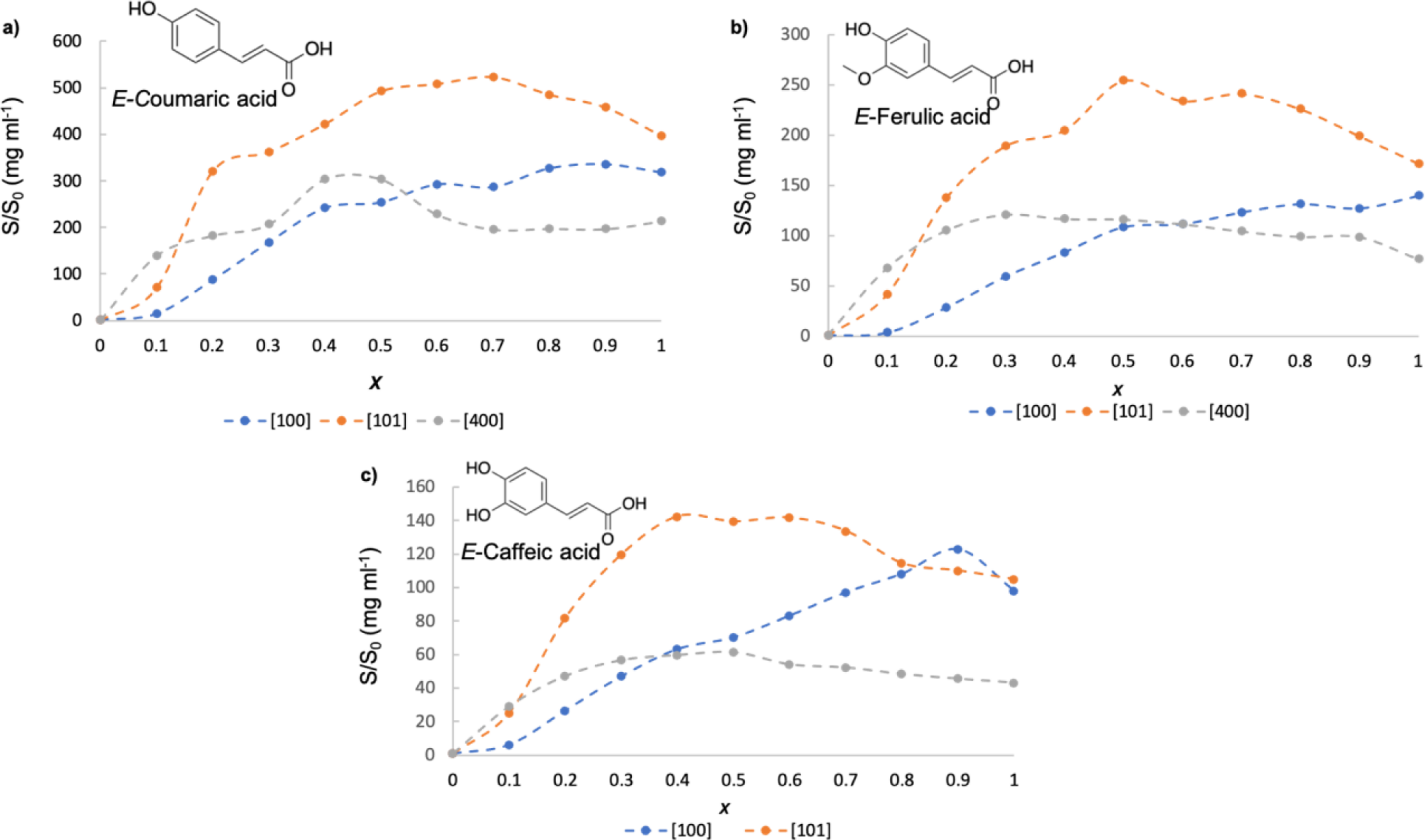
Effect of glycerol ether (**100**, **101**, and **400**) molar fraction on the solubility of (a) coumaric, (b) ferulic, and (c) caffeic acids in aqueous solutions.

In the case of using **100** monoether (Figure 9: blue dashed line), an almost linear increase of the solubility is observed when increasing the molar ratio of the glycerol-derived solvent in all cases. Only a hydrotropic effect is observed in the case of caffeic acid at *X* = 0.9, with an increase of 20% in the solubility compared to pure **100**.

In the case of using more hydrophobic solvents such as **101** and **400**, the maximum solubility is observed for all three solutes at a molar fraction of less than one. It is noticeable that with **101**, the solubility in the mixture compared to the solubility in water can be increased 500-fold in the case of coumaric acid, 250-fold in the case of ferulic acid, and 140-fold in the case of caffeic acid. Thus, solubility values were close to those obtained with EGMME (Figure 6 and Tables S13–S15).

Having a close look at the more diluted region (0 < *X* < 0.1), the greater increase of solubility is provided by solvent **400**, followed by solvent **101**, in all cases. This behavior is in line with that previously described for gallic and syringic acids, in which the hydrophobicity of the solvent was the driving force for the hydrotropic effect at lower hydrotrope concentrations.^33^ This increase in the diluted region is of great interest for considering these solvents as solubilizing media or in pharmaceutical formulations.

Above this, when comparing the hydrotropy effect for ferulic acid with our solvents and the ones previously studied by Silva et al.^30^ with renewable solvents such as γ-valerolactone, ethyl lactate, or cyrene (Figure 10), it can be observed that **101** diether provided the greatest hydrotropic effect, with a 268-fold increase in solubility over water, while in the diluted region, **400** and γ-valerolactone seem to be the best hydrotropes.

**Figure 10:**
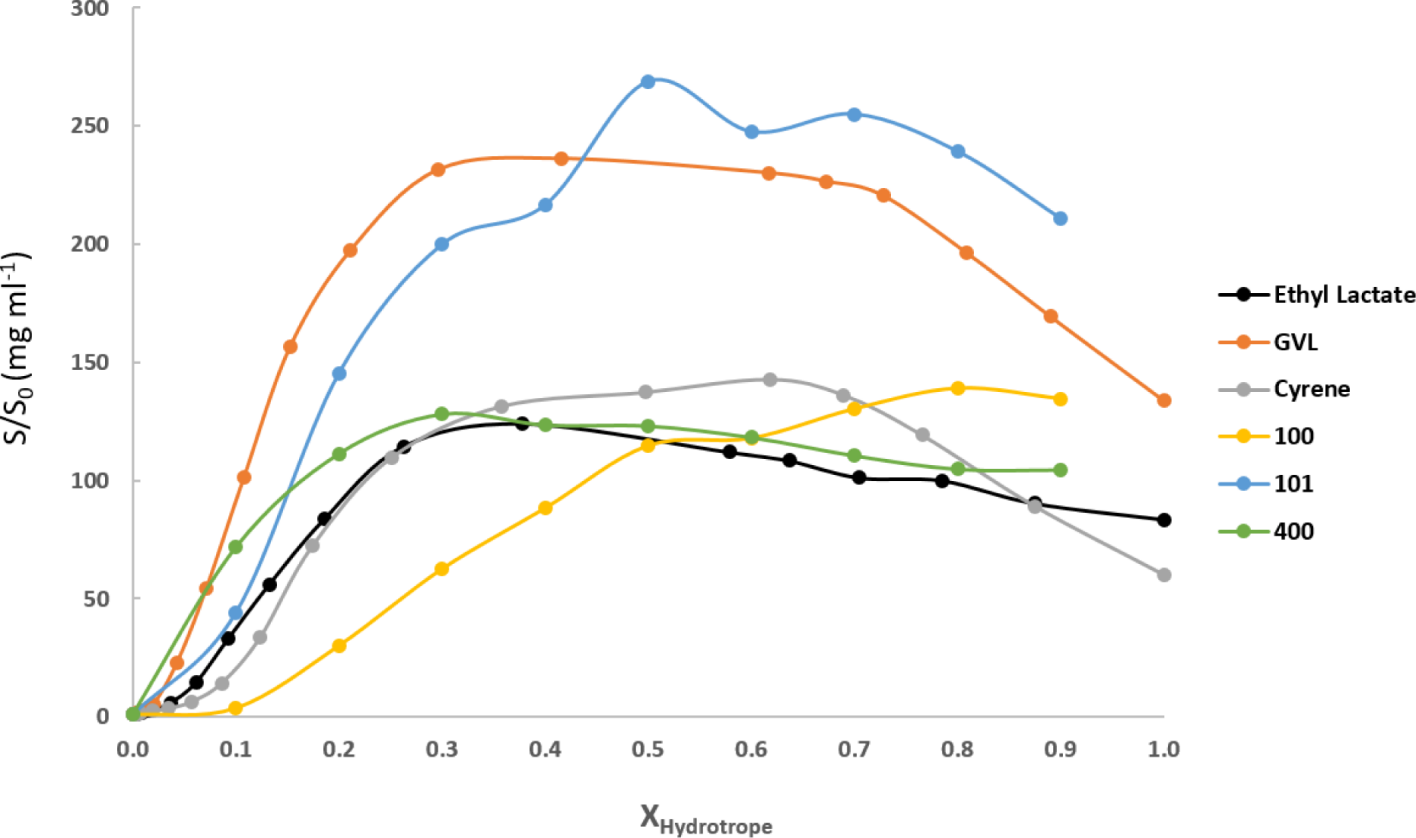
Comparison of the hydrotropic effect on ferulic acid solubilization in glycerol-derived ethers and biobased solvents.^30^

## Conclusions

Glycerol-derived ethers, particularly methyl ethers (e.g., 100 and 101), were found to significantly enhance the solubility of hydroxycinnamic acids, outperforming conventional fossil-sourced solvents such as ethylene glycol and propylene glycol. An influence of the nature of the substituent has been observed, with methyl derivatives providing the best solubility values in all cases, while increasing the length of the substituents or the presence of fluorine atoms in the solvents decreased the solubility. Solubility trends were influenced by the hydrogen-bond donor (HBD) capacity and polarity-polarizability of the solvents, as indicated by experimental results and COSMO-RS modeling. In aqueous mixtures, glycerol ethers exhibited hydrotropic effects, enhancing solubility at low solvent concentrations. **101** diether showed the most significant solubility increase across all acids, especially in dilute regions. Computational predictions aligned with experimental data, identifying **101** diether as the most effective solvent. Trends related to the alkyl chain length, substitution patterns, and fluorination were confirmed. COSMO-RS successfully predicted solubility trends and proved to be useful for solvent design selection using a low time-consuming relative screening method, despite some under- or overestimation of solubility values due to the neglect of spatial information that is worsened when using fast noniterative screening methods. Nevertheless, we observed that a more precise solubility estimation can be achieved by applying SLE settings. Finally, we can conclude that glycerol-derived ethers provide a sustainable, renewable, and low-toxicity alternative to conventional solvents for the solubilization of hydroxycinnamic acids, aligning with green chemistry principles. These solvents hold promise for applications in pharmaceutical formulations, cosmetic products, and biomass processing, where hydroxycinnamic acids play a critical role.

## Supplementary Material



## Notes

List of abbreviations and acronyms, characterization of the glycerol-derived solvents, HPLC calibrations of the studied acids, solubility values, Cartesian coordinates for optimized geometries in Gaussian09, and comparison between experimental and COSMO-RS-calculated solubilities (PDF)
