# Supplementary material for: Eco-Friendly Solvents for Bioactives: Solubilization of Hydroxycinnamic Acids in Glycerol-Derived Ethers
Source: ACS Sustain Chem Eng. 2025 Jun 5;13(23):8556–66. doi: 10.1021/acssuschemeng.5c00269 (PMC12175220; doi:10.1021/acssuschemeng.5c00269)
Supplement: Supplementary file 1 [file sc5c00269_si_001.pdf]

SUPPORTING INFORMATION

Eco-Friendly Solvents for Bioactives: Solubilization of Hydroxycinnamic Acids in Glycerol Derived Ethers

Sara Gracia-Barberán[a,b]*, María Lanau*[b]*, Alejandro Leal-Duaso*[a,b]*, Pilar López* Ram de Viu[a,b]*, Ana M. Mainar*[c]*, José A. Mayoral* [a,b]*, Elísabet Pires**[, a,b]

[a]Departamento de Química Orgánica, Facultad de Ciencias, Universidad de Zaragoza, Calle Pedro Cerbuna, 12. E-500009, Zaragoza, Spain. E-mail:epires@unizar.es

[b]Instituto de Sintesis Química y Catálisis Homogénea (ISQCH-CSIC), Facultad de Ciencias, Universidad de Zaragoza. Calle Pedro Cerbuna, 12. E-50009, Zaragoza, Spain.

[c] *GATHERS Group, Aragón Institute of Engineering Research (I3A), Universidad de Zaragoza, c/. Mariano Esquillor s/n, 50018 Zaragoza, Spain*

TABLE OF CONTENTS

1. **List of abbreviations and acronyms** **S1**
2. **Characterization of the glycerol-derived solvents** **S2**
3. **HPLC calibrations of the studied acids** **S15**
4. **Solubility values** **S17**
5. **Cartesian coordinates for optimized geometries in Gaussian09** **S25**
6. **Comparison between experimental and COSMO-RS-calculated solubilities** **S29**
7. **^{1}H-NMR study of solute-solvent interactions in saturated solutions**

1. List of abbreviations and acronyms

**100**	3-Methoxypropane-1,2-diol
**200**	3-Ethoxypropane-1,2-diol
**300**	3-Propoxypropane-1,2-diol
**3i00**	3-Isopropoxypropane-1,2-diol
**3F00**	3-(2,2,2-Trifluoroethoxy)propane-1,2-diol
**400**	3-Butoxypropane-1,2-diol
**101**	1,3-Dimethoxypropan-2-ol
**202**	1,3-Diethoxypropan-2-ol
**303**	1,3-Dipropoxypropan-2-ol
**3i03i**	1,3-Diisopropoxypropan-2-ol
**3F03F**	1,3-Bis(2,2,2-trifluoroethoxy)propan-2-ol
**404**	1,3-Dibutoxypropan-2-ol
**111**	1,2,3-Trimethoxypropane
**PEG**	Polyethylene glycol
**EG**	Ethylene glycol
**PG**	Propylene glycol
**EGMME**	Ethylene glycol monomethylether
**EGDME**	Ethylene glycol monomethylether

2. Characterization of the glycerol-derived solvents

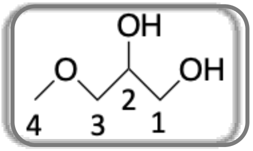

3-Methoxypropane-1,2-diol [100], ^{1}H NMR (400 MHz, [d$_6$]DMSO, 25 ºC): δ 4.61 (d, 1H, J = 5.1 Hz, OH$_2$), 4.46 (t, 1H, J = 5.7 Hz, OH$_1$), 3.56 (sext, 1H, J = 5.2 Hz, H$_2$), 3.26-3.36 (m, 3H, H$_1$, H$_{3a}$), 3.23 (s, 3H, H$_4$), 3.21 (dd, 1H, J_{gem} = 9.8 Hz, J = 6.0 Hz, H$_{3b}$). **^{13}C NMR** (100 MHz, [d$_6$]DMSO, 25 ºC): δ 74.2 (CH$_2$, C$_3$), 70.3 (CH, C$_2$), 62.9 (CH$_2$, C$_1$), 58.3 (CH$_3$, C$_4$).

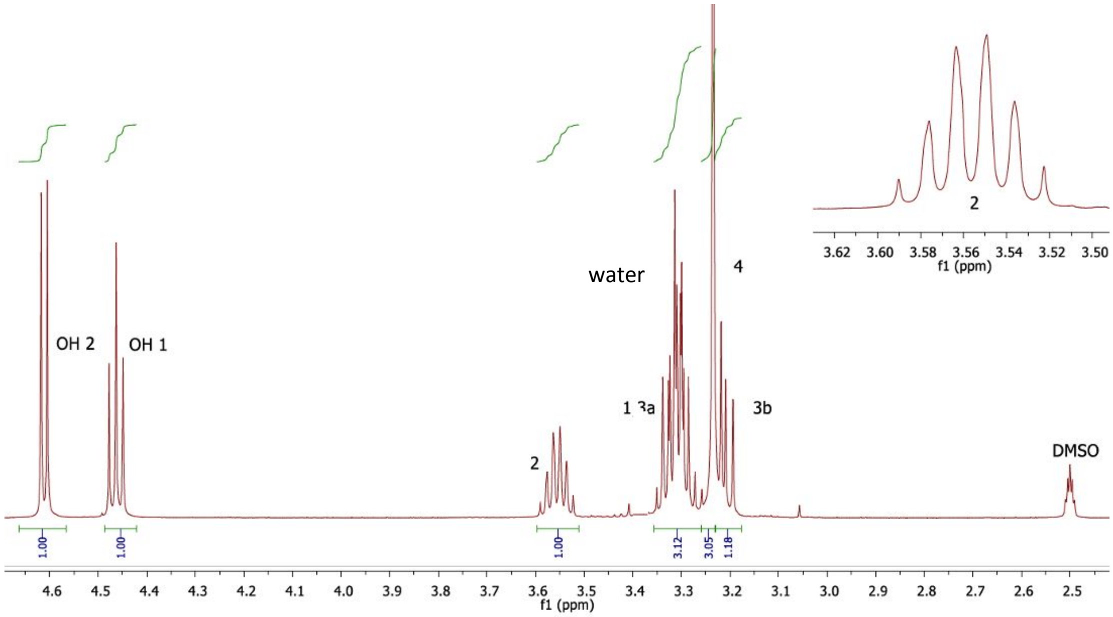

Figure S-1. ^{1}H NMR spectrum of 3-methoxypropane-1,2-diol [**100**]

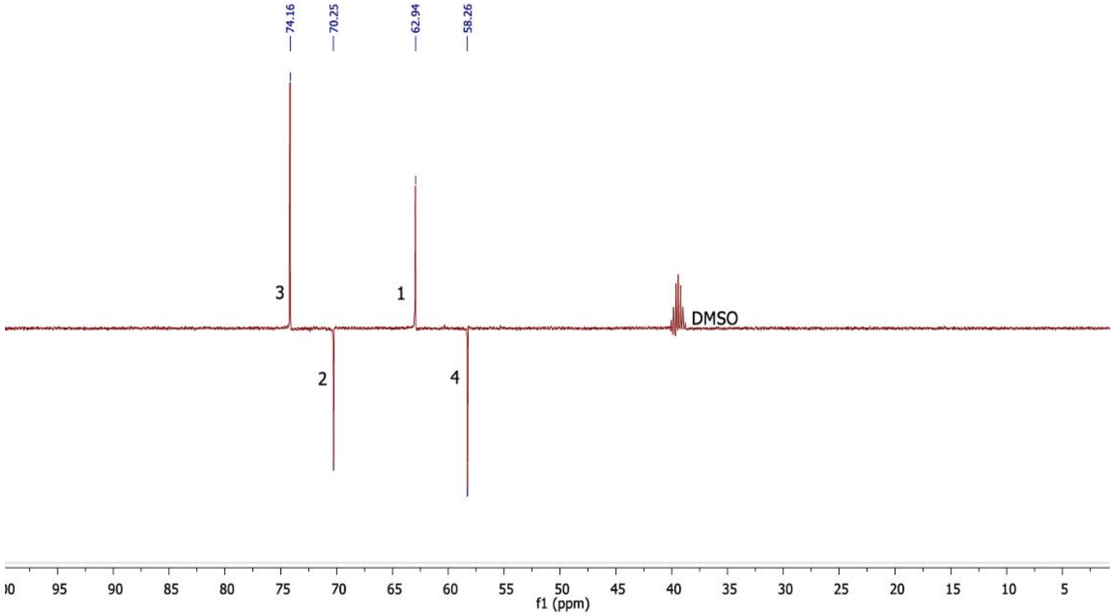

Figure S-2. ^{13}C NMR (APT) spectrum of 3-methoxypropane-1,2-diol [**100**]

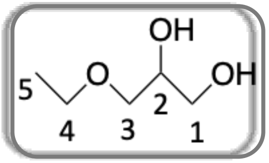

3-Ethoxypropane-1,2-diol [200], ^{1}H NMR (400 MHz, [d$_6$]DMSO, 25 ºC): δ 4.59 (d, 1H, *J* = 5.1 Hz, OH_2), 4.45 (t, 1H, *J* = 5.7 Hz, OH_1), 3.55 (sext, 1H, *J* = 5.3 Hz, H_2), 3.42 (q, 2H, *J* = 7.0 Hz, H_4), 3.21-3.37 (m, 4H, H_1, H_{3a}, H_{3b}), 1.10 (t, 3H, *J* = 7.0 Hz, H_5). **^{13}C NMR** (100 MHz, [d$_6$] DMSO, 25 ºC): δ 72.3 (CH_2, C_3), 70.8 (CH, C_2), 66.0 (CH_2, C_4), 63.4 (CH_2, C_1), 15.4 (CH_3, C_5).

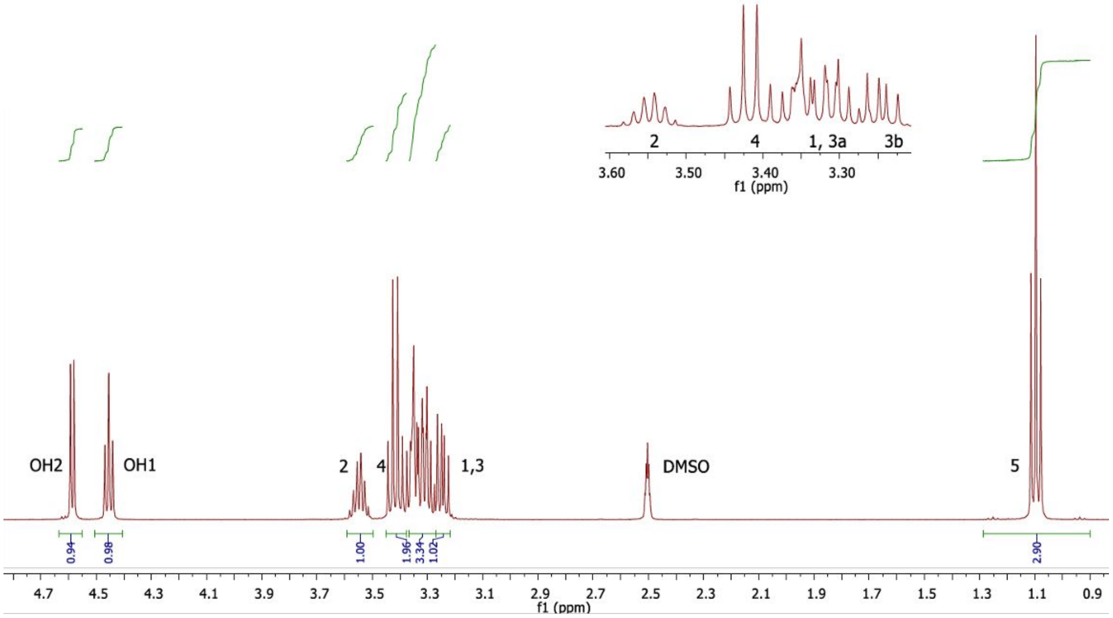

Figure S-3. ^{1}H NMR spectrum of 3-ethoxypropane-1,2-diol [**200**]

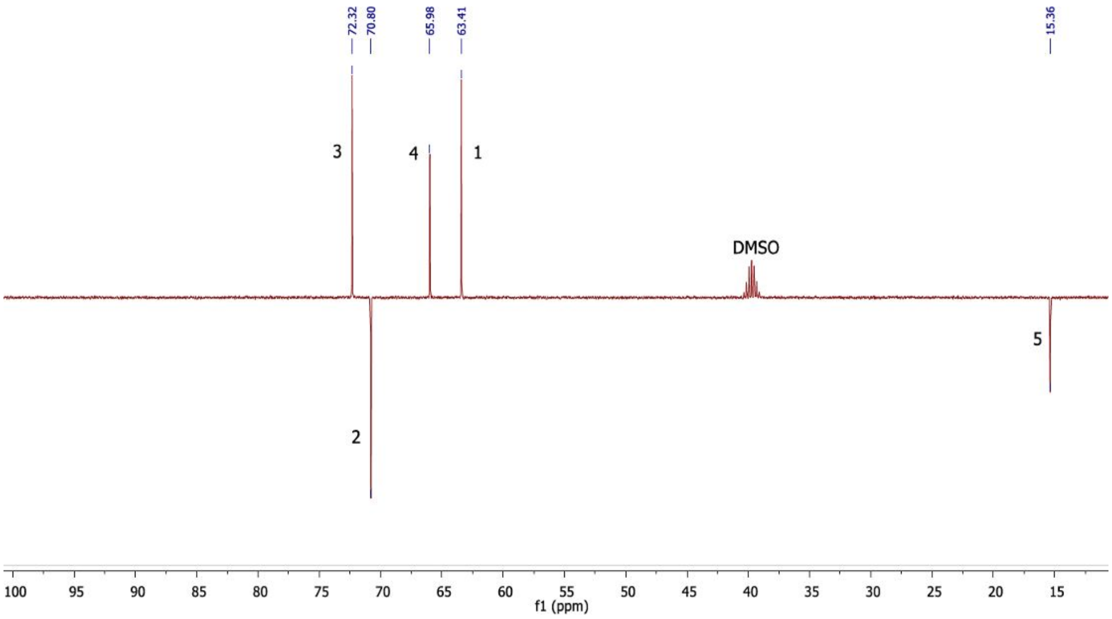

Figure S-4. ^{13}C NMR (APT) spectrum of 3-ethoxypropane-1,2-diol [**200**]

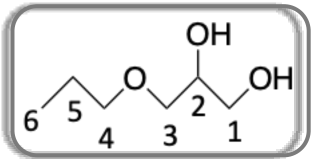

3-propoxypropane-1,2-diol [300], ^{1}H NMR (400 MHz, [d$_6$]DMSO, 25 ºC): δ 4.57 (d, 1H, J = 5.1 Hz, OH$_2$), 4.44 (t, 1H, J = 5.7 Hz, OH$_1$), 3.55 (sext, 1H, J = 5.3 Hz, H$_2$), 3.32 (t, 2H, J = 7.3 Hz, H$_4$), 3.27-3.38 (m, 3H, H$_1$, H$_{3a}$), 3.25 (dd, 1H, J_{gem} = 9.8 Hz, J = 6.0 Hz, H$_{3b}$), 1.48 (sext, 2H, J = 7.3 Hz, H$_5$), 0.85 (t, 3H, J = 7.4 Hz, H$_6$). **^{13}C NMR** (100 MHz, [d$_6$]DMSO, 25 ºC): δ 72.2 (CH$_2$, C$_3$) 72.1 (CH$_2$, C$_4$), 70.5 (CH, C$_2$), 63.2 (CH$_2$, C$_1$), 22.5 (CH$_2$, C$_5$), 10.5 (CH$_3$, C$_6$).

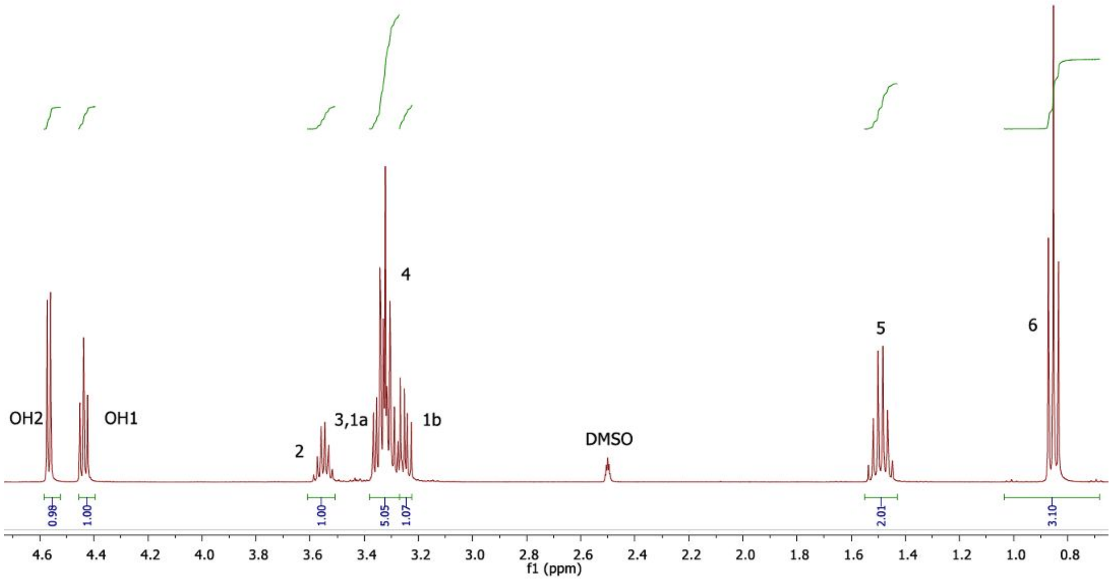

Figure S-5. ^{1}H NMR spectrum of 3-propoxypropane-1,2-diol [**300**]

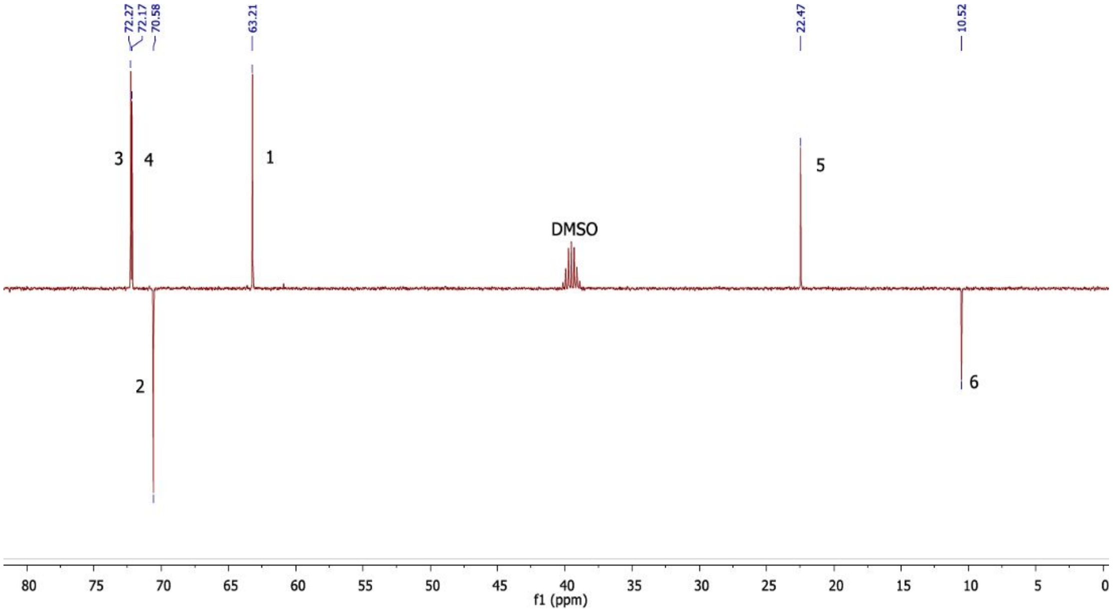

Figure S-6. ^{13}C NMR (APT) spectrum of 3-propoxypropane-1,2-diol [**300**]

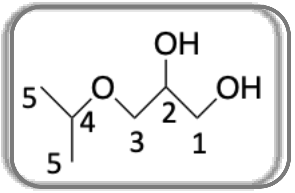

3-Isopropoxypropane-1,2-diol [3i00], ^{1}H NMR (400 MHz, [d_6]DMSO, 25 ºC): δ 4.52 (d, 1H, *J* = 5.0 Hz, OH_2), 4.42 (t, 1H, *J* = 5.7 Hz, OH_1), 3.46-3.56 (m, 2H, H_2, H_4), 3.26-3.38 (m, 3H, H_1, H_{3a}), 3.23 (dd, 1H, J_{gem} = 9.6 Hz, *J* = 6.0 Hz, H_{3b}), 1.07 (d, 6H, *J* = 6.1 Hz, H_5). **^{13}C NMR** (100 MHz, [d_6]DMSO, 25 ºC): δ 71.0 (CH, $C_{2\text{ ó }4}$), 71.0 (CH, $C_{4\text{ ó }2}$), 69.7 (CH_2, C_3), 63.3 (CH_2, C_1), 22.1 (CH_3, C_5).

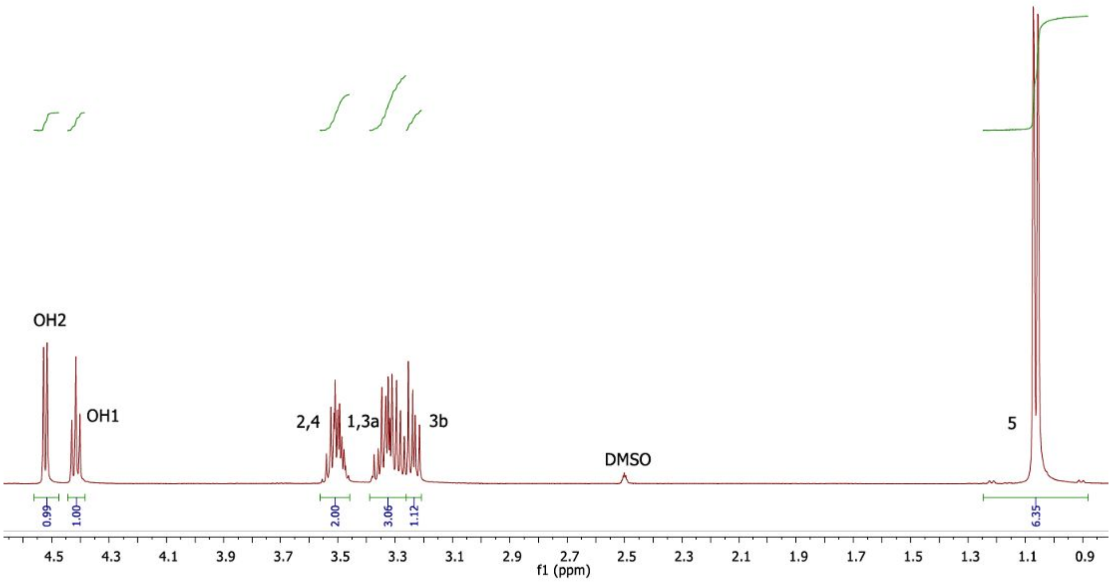

Figure S-7. ^{1}H NMR spectrum of 3-isopropoxypropane-1,2-diol [**3i00**]

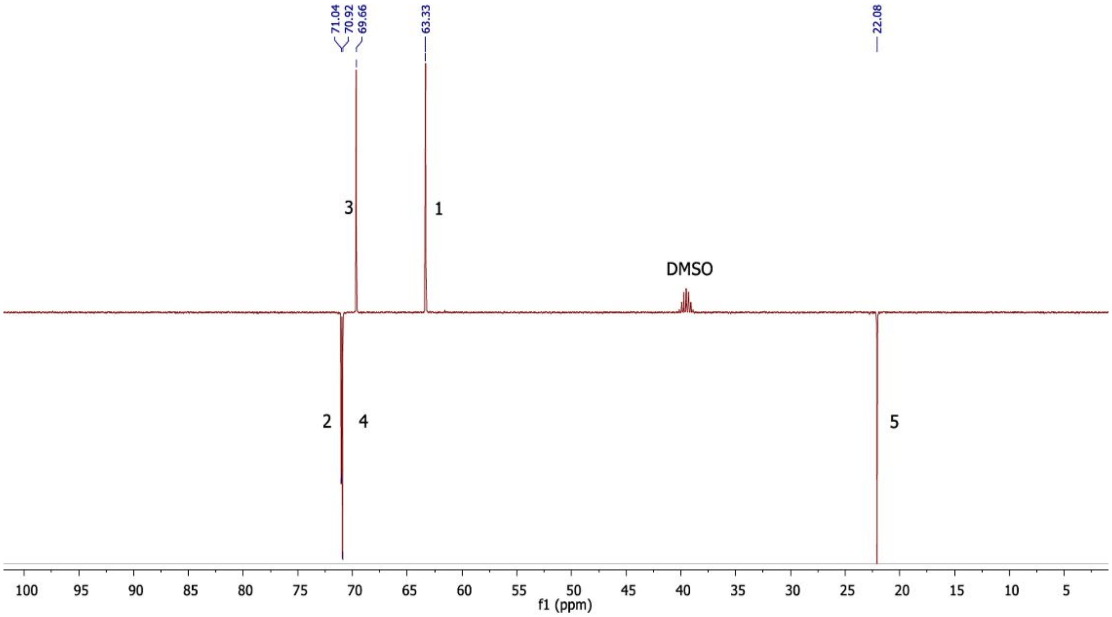

Figure S-8. ^{13}C NMR (APT) spectrum of 3-isopropoxypropane-1,2-diol [**3i00**]

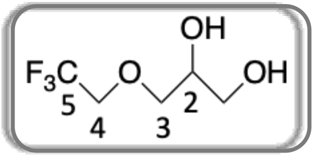

3-(2,2,2-Trifluoroethoxy)propane-1,2-diol [3F00],^{1}H NMR (400 MHz, [d_6]DMSO, 25 ºC): δ 4.80 (d, 1H, J = 5.2 Hz, OH_2), 4.57 (t, 1H, J = 5.6 Hz, OH_1), 4.05 (q, 2H, J = 9.4 Hz, H_4), 3.57-3.64 (m, 2H, H_2, H_{3a}), 3.45-3.52 (m, 1H, H_{3b}), 3.32 (dd, 2H, J = 5.6 Hz, 5.7 Hz, H_1). **^{13}C NMR** (100 MHz, [d_6]DMSO, 25 ºC): δ 124.6 (q, CF_3, J = 279.6 Hz, C_5), 73.9 (CH_2, C_3), 70.5 (CH, C_2), 67.7 (q, CH_2, J = 32.6 Hz, C_4), 62.7 (CH_2, C_1). **^{19}F NMR** (400 MHz, [d_6]DMSO, 25 ºC): δ –73.0 (t, CF_3, J = 9.4 Hz, F_5).

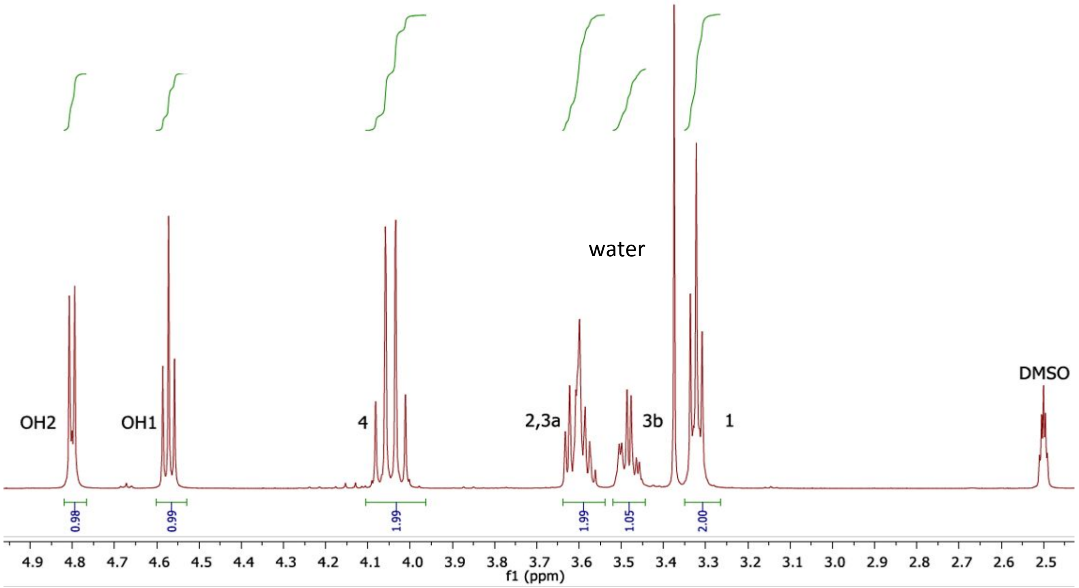

Figure S-9. ^{1}H NMR spectrum of 3-(2,2,2trifluoroethoxy)propane-1,2-diol [**3F00**]

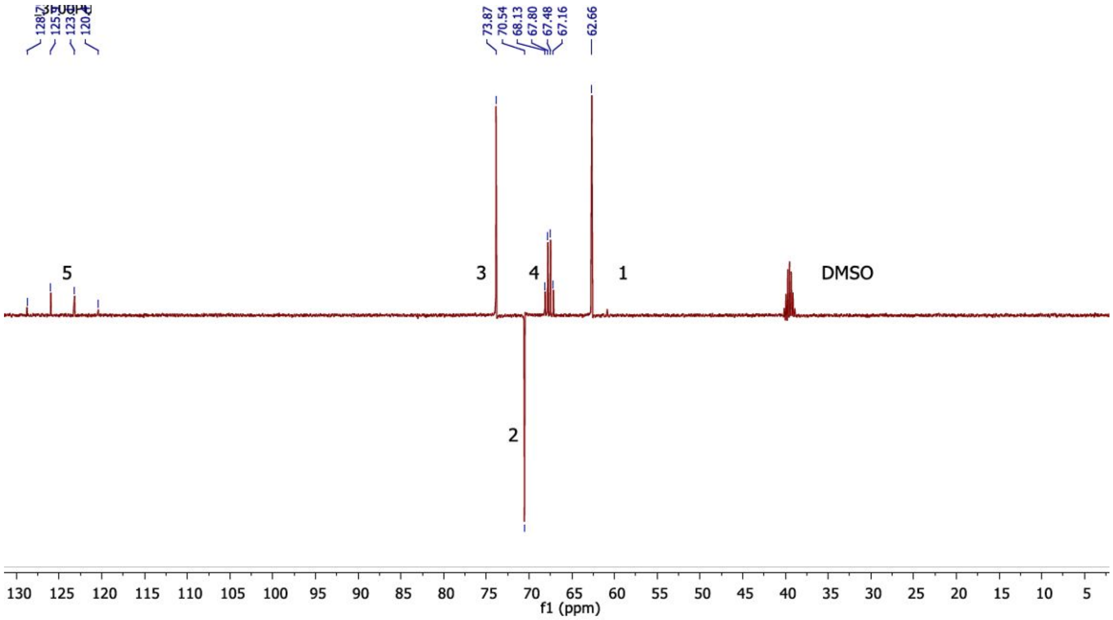

Figure S-10. ^{13}C NMR (APT) spectrum of 3-(2,2,2trifluoroethoxy)propane-1,2-diol [**3F00**]

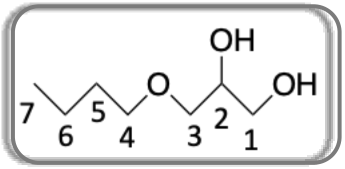

3-Butoxypropane-1,2-diol [400], ^{1}H NMR (400 MHz, [d$_6$]DMSO, 25 ºC): δ 4.54 (d, 1H, J = 5.1 Hz, OH$_2$), 4.41 (t, 1H, J = 5.7 Hz, OH$_1$), 3.53 (sext, 1H, J = 5.2 Hz, H$_2$), 3.34 (t, 2H, J = 6.7 Hz, H$_4$), 3.24-3.32 (m, 3H, H$_1$, H$_{3a}$), 3.22 (dd, 1H, J_{gem} = 9.8 Hz, J = 6.0 Hz, H$_{3b}$), 1.44 (quint, 2H, J = 7.6 Hz, H$_5$), 1.29 (sext, 2H, J = 7.3 Hz, H$_6$), 0.85 (t, 3H, J = 7.4 Hz, H$_7$). **^{13}C NMR** (100 MHz, [d$_6$]DMSO, 25 ºC): δ 72.3 (CH$_2$, C$_3$), 70.6 (CH, C$_2$), 70.2 (CH$_2$, C$_4$), 63.2 (CH$_2$, C$_1$), 31.4 (CH$_2$, C$_5$), 18.9 (CH$_2$, C$_6$), 13.8 (CH$_3$, C$_7$).

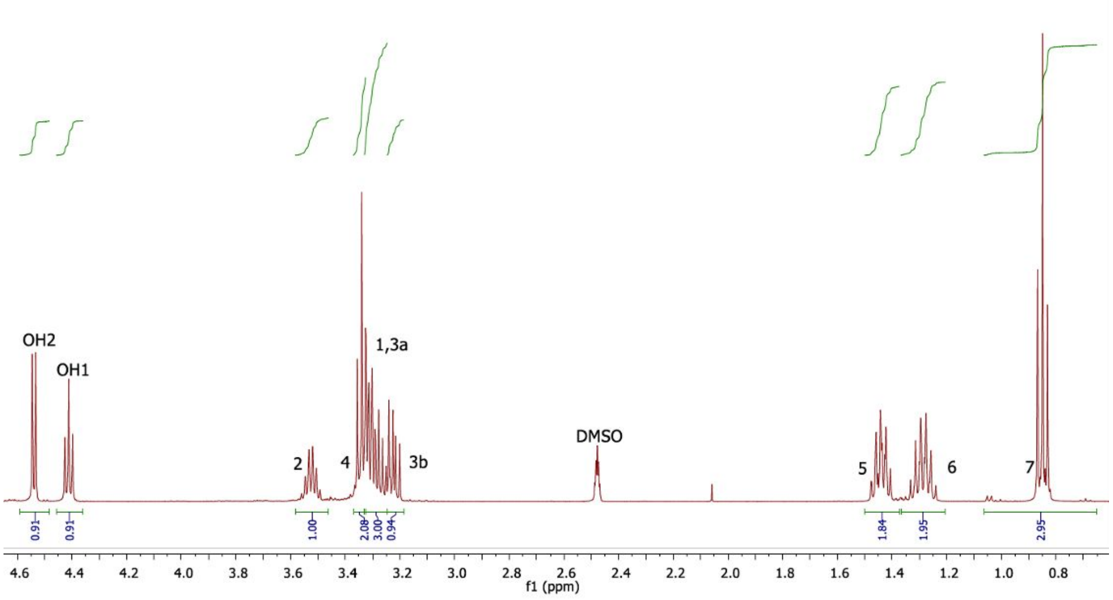

Figure S-11. ^{1}H NMR spectrum of 3-Butoxypropane-1,2-diol [**400**]

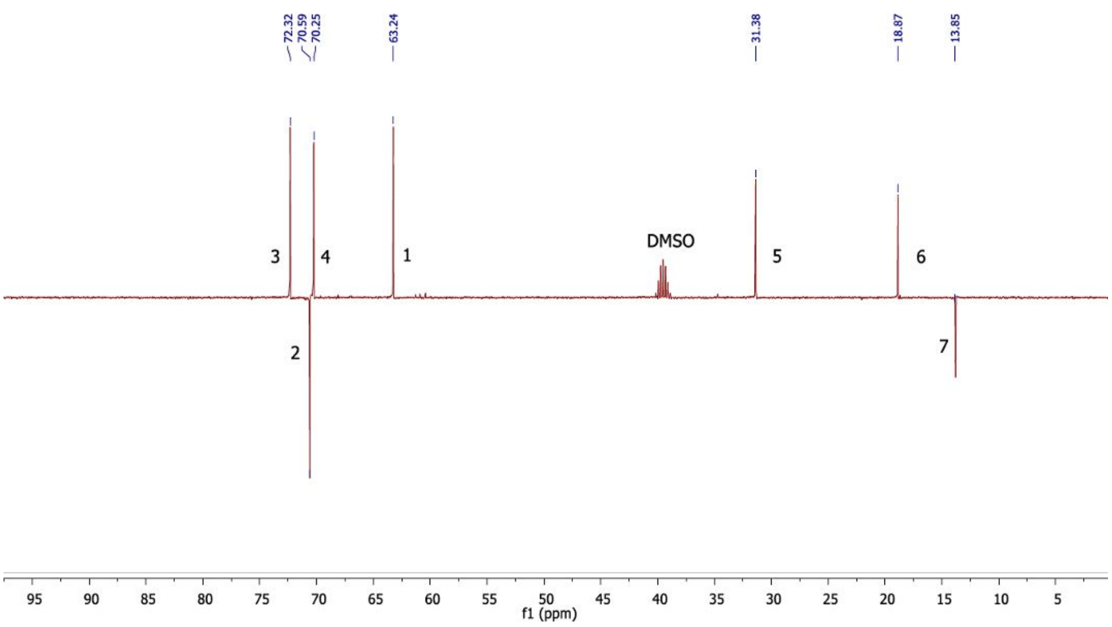

Figure S-12. ^{13}C NMR (APT) spectrum of 3-Butoxypropane-1,2-diol [**400**]

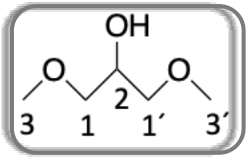

1,3-Dimethoxypropan-2-ol [101], ^{1}**H NMR** (400 MHz, [d$_6$]DMSO, 25 ºC): δ 4.79 (d, 1H, J = 5.2 Hz, OH$_2$), 3.70 (sext, 1H, J = 5.1 Hz, H$_2$), 3.28 (dd, 2H, J_{gem} = 9.8 Hz, J = 5.0 Hz, H$_{1a}$, H$_{1'a}$), 3.23 (dd, 2H, J_{gem} = 9.9 Hz, J = 5.6 Hz, H$_{1b}$, H$_{1'b}$), 3.24 (s, 6H, H$_3$, H$_{3'}$). ^{13}**C NMR** (100 MHz, [d$_6$]DMSO, 25 ºC): δ 74.2 (CH$_2$, C$_{1/1'}$), 68.2 (CH, C$_2$), 58.4 (OCH$_3$, C$_{3/3'}$).

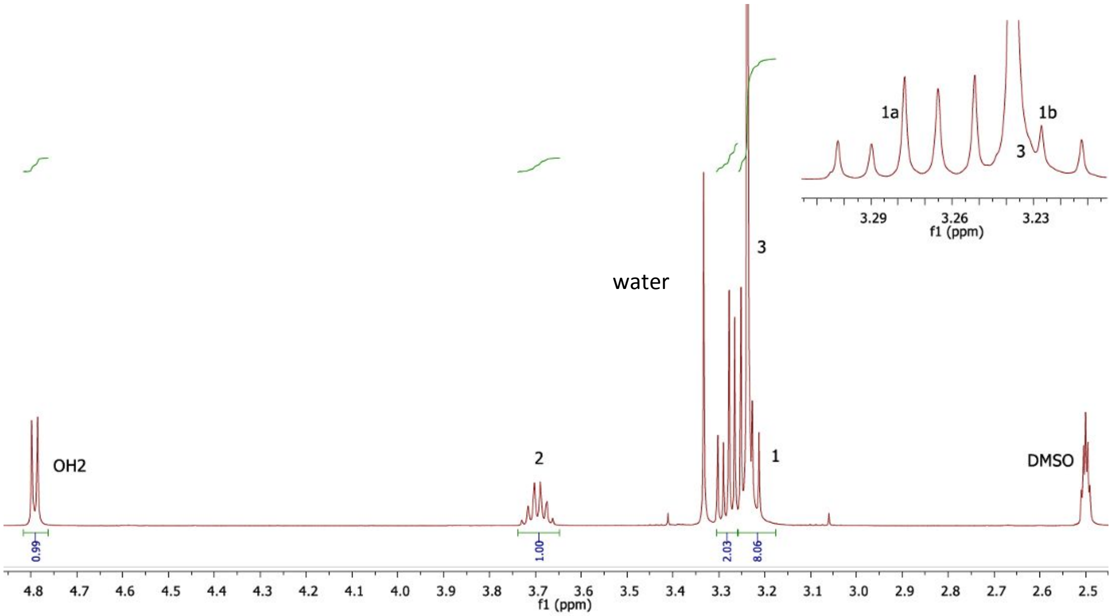

Figure S-13. ^{1}H NMR spectrum of 1,3-Dimethoxypropan-2-ol [**101**]

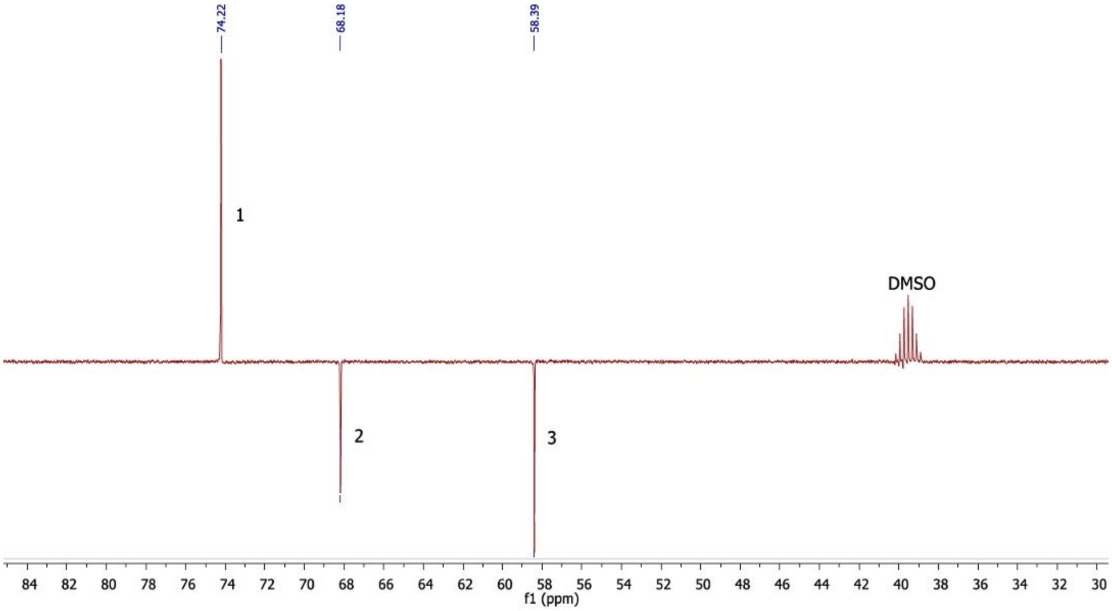

Figure S-14. ^{13}C NMR (APT) spectrum of 1,3-Dimethoxypropan-2-ol [**101**]

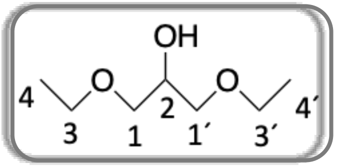

1,3-Diethylpropan-2-ol [202], ^{1}**H NMR** (400 MHz, [d$_6$]DMSO, 25 ºC): δ 4.72 (d, 1H, J = 5.2 Hz, OH_2), 3.67 (sext, 1H, J = 5.1 Hz, H_2), 3.42 (c, 4H, J = 7,0 Hz, H_3, H_3), 3.32 (dd, 2H, J_{gem} = 9.8 Hz, J = 5.0 Hz, H_{1a}, $H_{1'a}$), 3,26 (dd, 2H, J_{gem}=9,9 Hz, J = 5,9 Hz, H_{1b}, $H_{1'b}$), 1,09 (t, 6H, J = 7,0 Hz, H_4, $H_{4'}$). ^{13}**C NMR** (100 MHz, [d$_6$]DMSO, 25 ºC): δ 72,2 (CH_2, $C_{1/1'}$), 68.6 (CH, C_2), 65,8 (CH_2, $C_{3/3'}$), 15,1 (CH_3, $C_{4/4'}$)

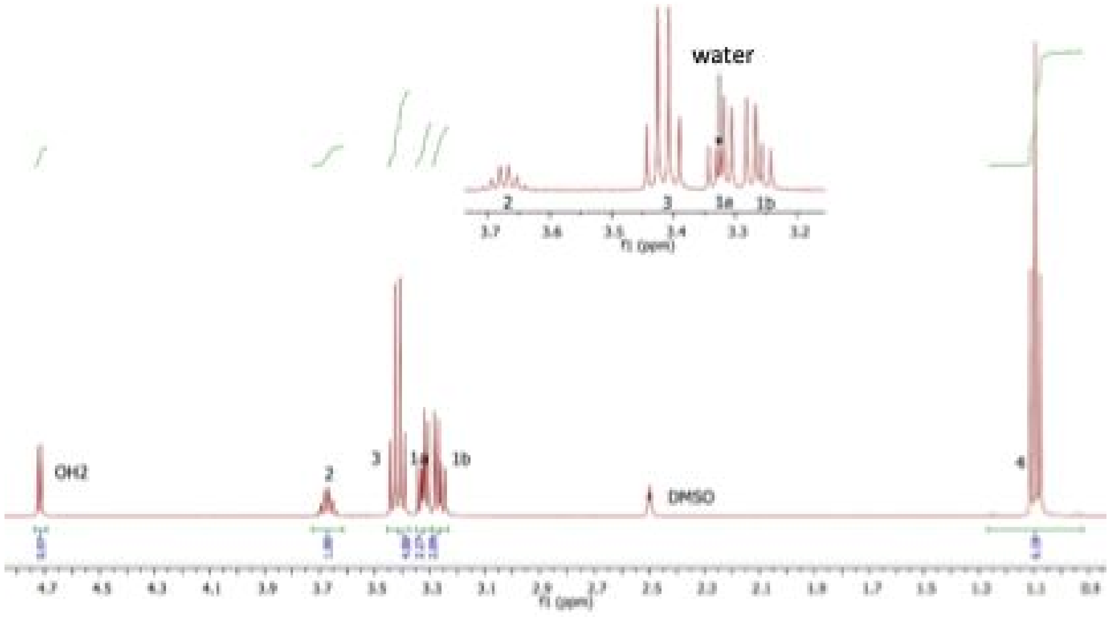

Figure S-15. ^{1}H NMR spectrum of 1,3-Diethylpropan-2-ol [**202**]

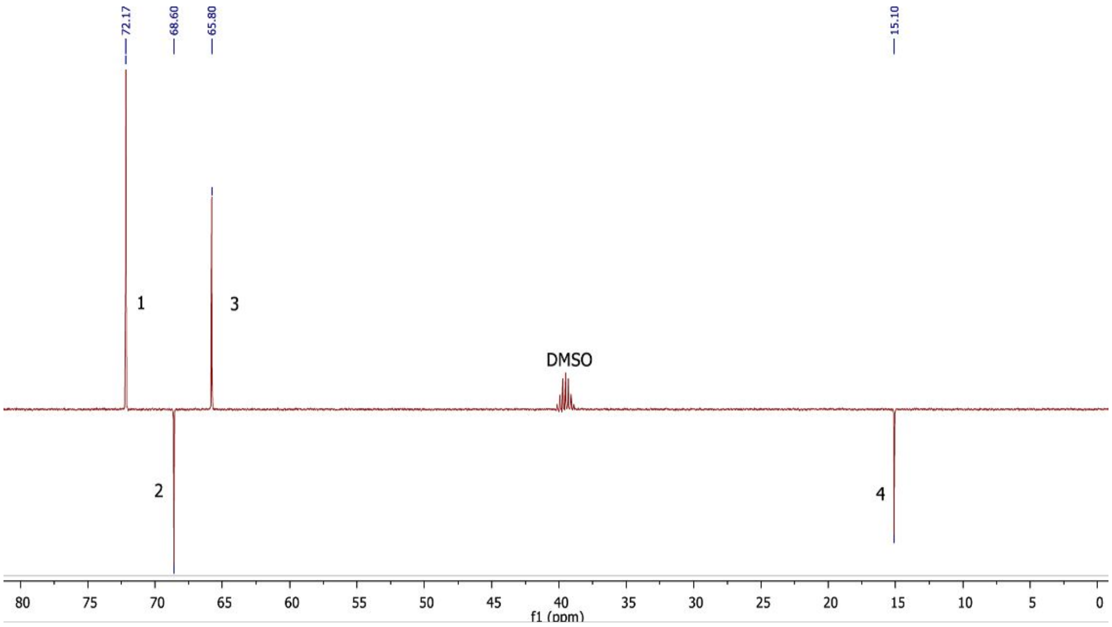

Figure S-16. ^{13}C NMR (APT) spectrum of 1,3-Diethylpropan-2-ol [**202**]

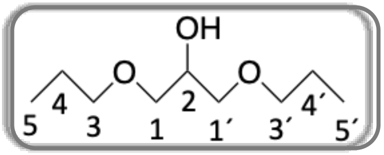

1,3-Dipropoxypropan-2-ol [303] ^{1}**H NMR** (400 MHz, [d$_6$]DMSO, 25 ºC): δ 4.69 (d, 1H, J = 5.2 Hz, OH_2), 3.68 (sext, 1H, J = 5.3 Hz, H_2), 3.33 (t, 4H, J = 6,6 Hz, H_3, H_3), 3.32 (dd, 2H, J_{gem} = 9.9 Hz, J = 5.1 Hz, H_{1a}, $H_{1'a}$), 3,27 (dd, 2H, J_{gem}=9,8 Hz, J = 5,8 Hz, H_{1b}, $H_{1'b}$), 1,49 (sx, 4H, J = 7,2 Hz, H_4, $H_{4'}$), 0,86 (t, 6 H, J = 7,4 Hz, H_5, $H_{5'}$) ^{13}**C NMR** (100 MHz, [d$_6$]DMSO, 25 ºC): δ 72,2 (CH_2, $C_{1/1'}$,$C_{3/3'}$), 68.6 (CH, C_2), 22,4 (CH_2, $C_{4/4'}$), 15,1 (CH_3, $C_{4/4'}$)

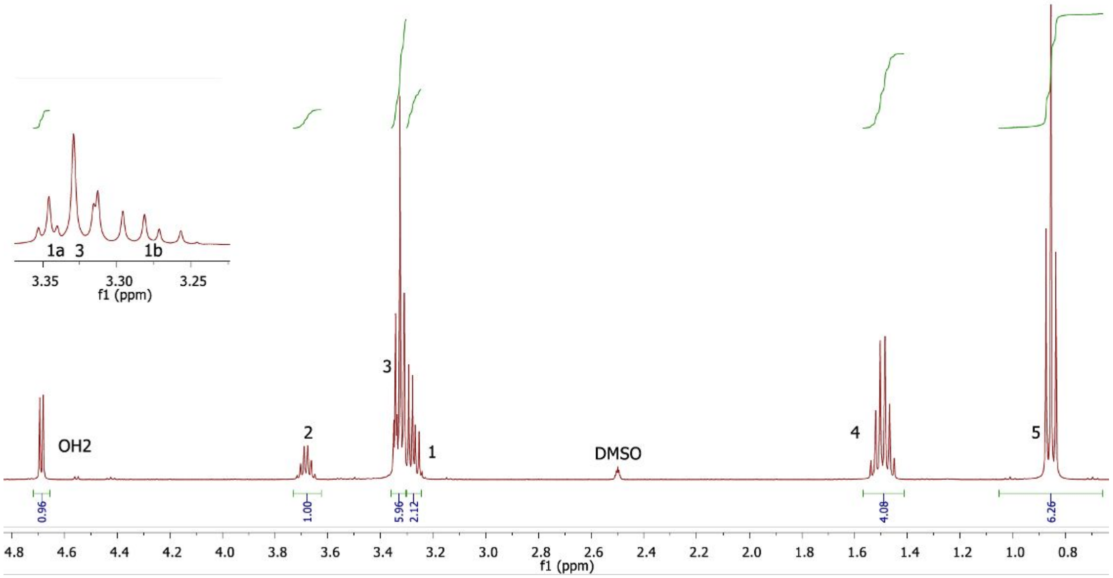

Figure S-17. ^{1}H NMR spectrum of 1,3-Dipropoxypropan-2-ol [**303**]

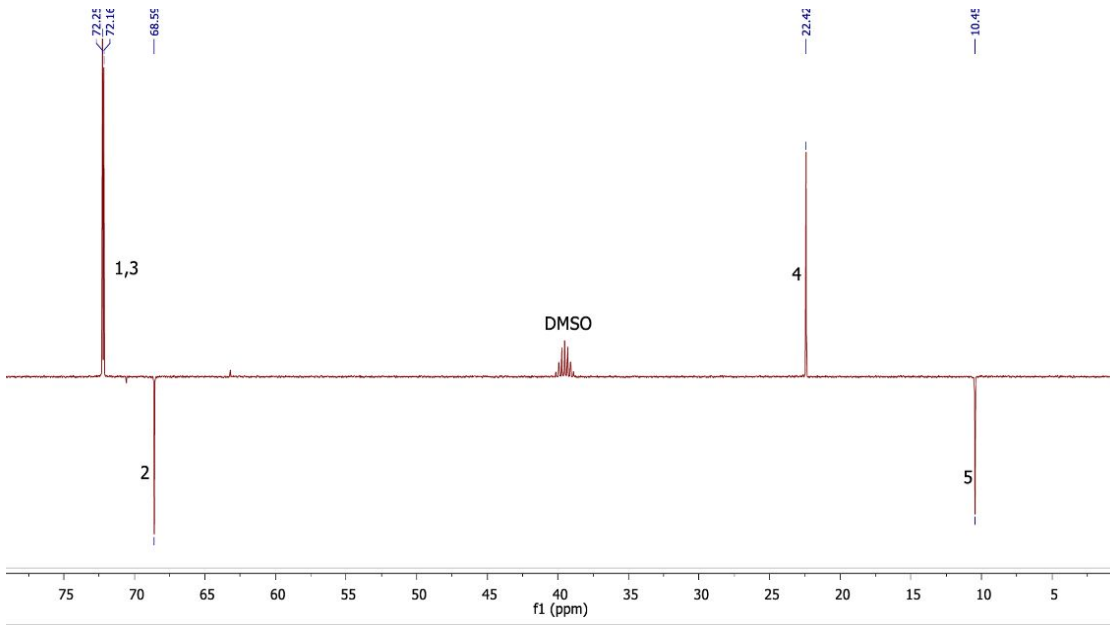

Figure S-18. ^{13}C NMR (APT) spectrum of 1,3-Dipropoxypropan-2-ol [**303**]

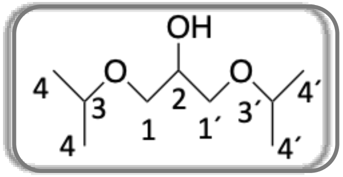

1,3-Diisopropoxypropan-2-ol [3i03i] ^{1}H NMR (400 MHz, [d$_6$]DMSO, 25 ºC): δ 4.61 (d, 1H, *J* = 5.2 Hz, OH$_2$), 3.58 (sx, 1H, *J* = 5.5 Hz, H$_2$), 3.51 (sp, 2H, *J* = 6,1 Hz, H$_3$, H$_3$), 3.32 (dd, 2H, J_{gem} = 9.7 Hz, *J* = 5.1 Hz, H$_{1a}$, H$_{1'a}$), 3,25 (dd, 2H, J$_{gem}$=9,6 Hz, J = 5,8 Hz, H$_{1b}$, H$_{1'b}$), 1,07 (d, 12H, J = 76,1 Hz, H$_4$, H$_{4'}$) **^{13}C NMR** (100 MHz, [d$_6$]DMSO, 25 ºC): δ 71,0 (CH, C$_{3/3'}$), 69,6 (CH$_2$, C$_{1/1'}$), 69,2 (CH, C$_2$), 22,0 (CH$_3$, C$_{4/4'}$)

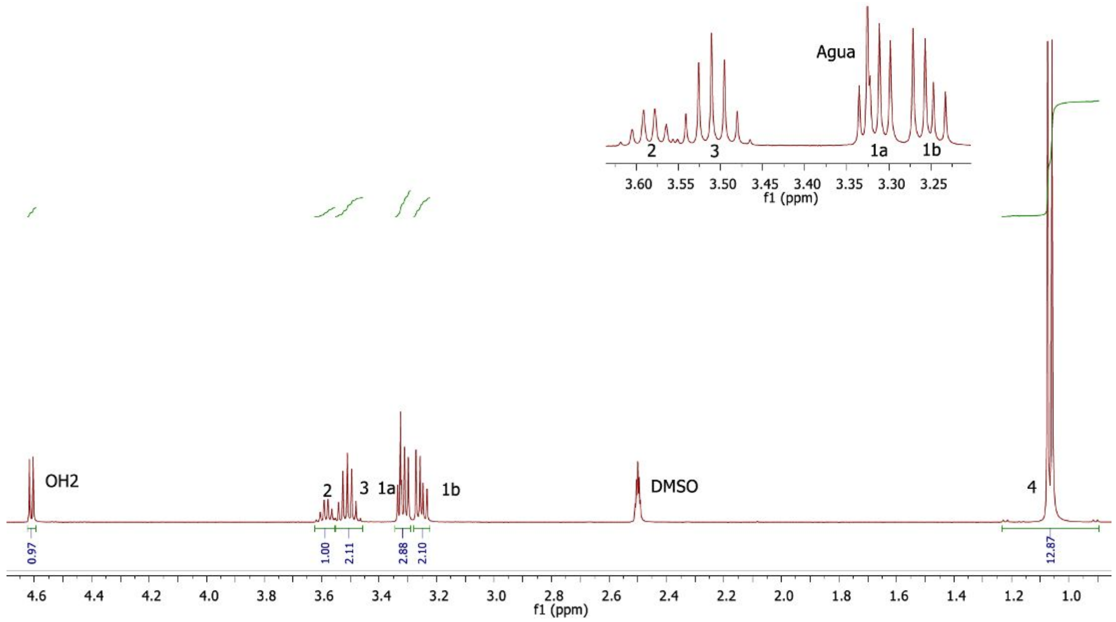

Figure S-19. ^{1}H NMR spectrum of 1,3-Diisopropoxypropan-2-ol [**3i03i**]

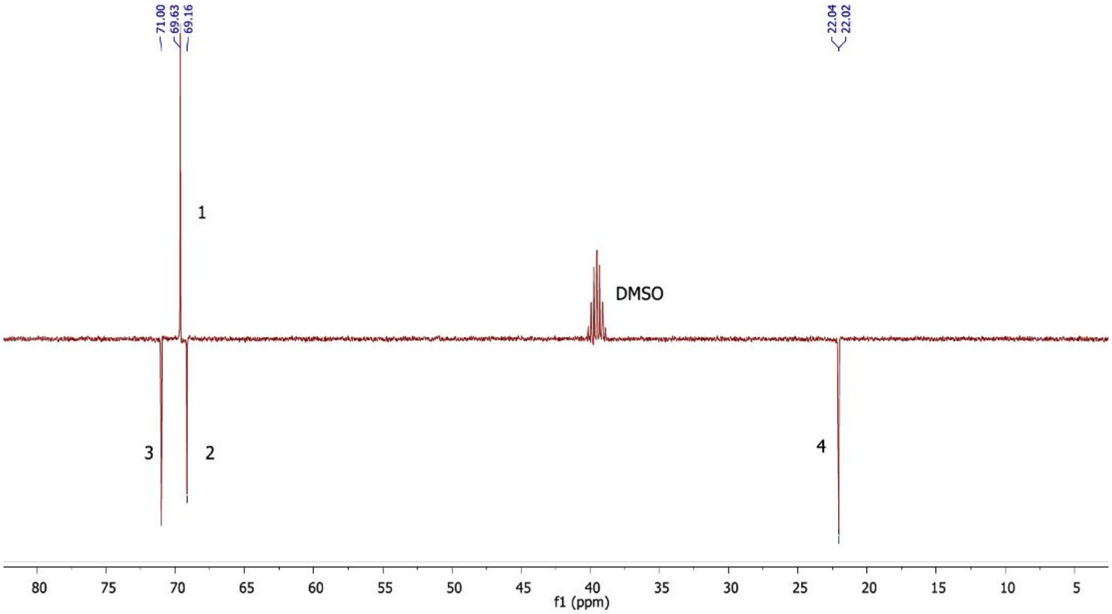

Figure S-20. ^{13}C NMR (APT) spectrum of 1,3-Diisopropoxypropan-2-ol [**3i03i**]

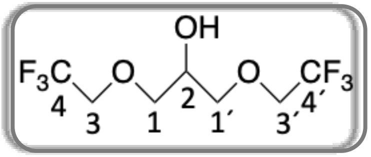

1,3-Bis(2,2,2-trifluoroethoxy)propan-2-ol [3F03F], ^{1}H NMR (400 MHz, [d$_6$]DMSO, 25 ºC): δ 5.13 (d, 1H, J = 5.3 Hz, OH$_2$), 4.07 (q, 4H, $J_{H\text{-}F}$ = 9.4 Hz, H$_3$, H$_{3'}$), 3,78 (sext, 1H, J = 5.3 Hz, H$_2$), 3.58 (dd, 2H, J_{gem} = 10.2 Hz, J = 4.8 Hz, H$_{1a}$, H$_{1'a}$), 3.53 (dd, 2H, J_{gem} = 10.2 Hz, J = 5,8 Hz, H$_{1b}$, H$_{1'b}$). **^{13}C NMR** (100 MHz, [d$_6$]DMSO, 25 ºC): δ 124.5 (q, CF$_3$, $J_{C\text{-}F}$ = 279.6 Hz, C$_{4/4'}$), 73.3 (CH$_2$, C$_{1/1'}$), 68.2 (CH, C$_2$), 67.6 (q, CH$_2$, $J_{C\text{-}F}$ = 32.6 Hz, C$_{3/3'}$). **^{19}F NMR** (400 MHz, [d$_6$]DMSO): δ –72.8 (t, CF$_3$, J = 9.4 Hz, C$_4$).

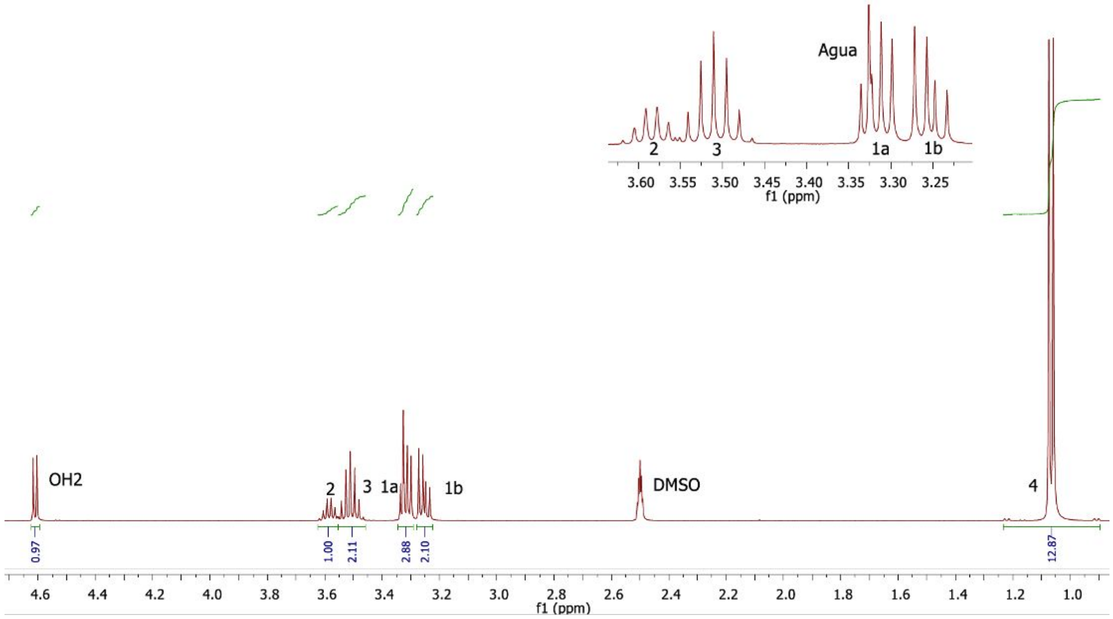

Figure S-21. ^{1}H NMR spectrum of 1,3-Bis(2,2,2-trifluoroethoxy)propan-2-ol [**3F03F**]

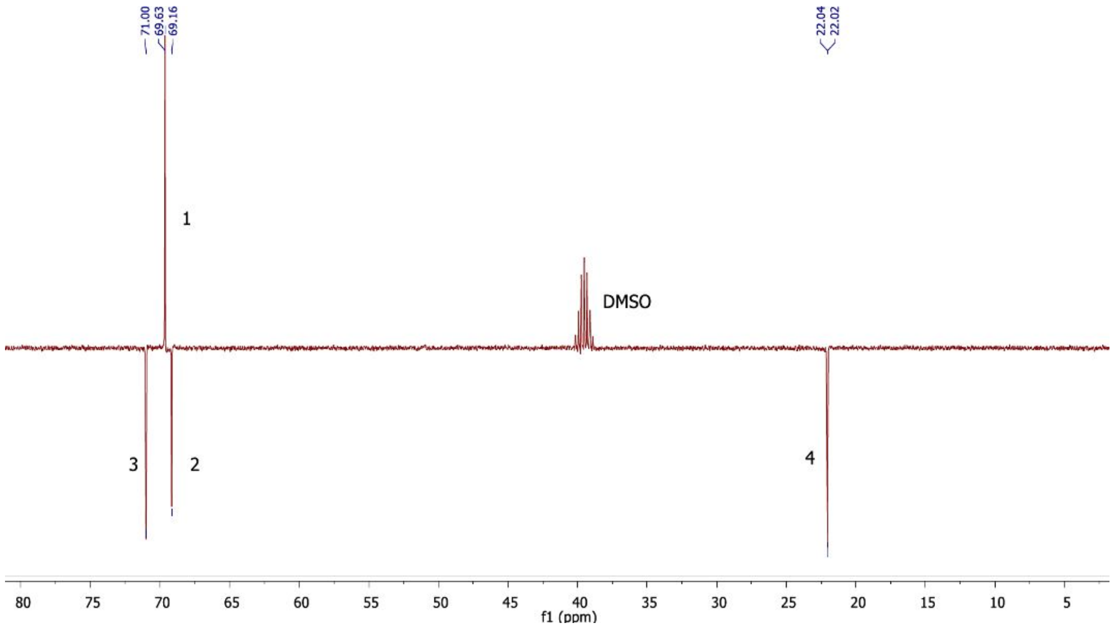

Figure S-22. ^{13}C NMR (APT) spectrum of 1,3-Bis(2,2,2-trifluoroethoxy)propan-2-ol [**3F03F**]

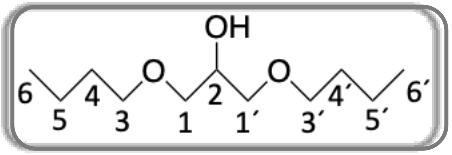

1,3-Dibutoxypropan-2-ol [404], ^{1}H NMR (400 MHz, [d_6]DMSO, 25 ºC): δ 4,69 (d, 1H, *J* = 5.0 Hz, OH_2), 3,67 (sx, 1H, *J* = 5,1 Hz, H_2), 3,36 (t, 4H, *J* = 6,5 Hz, H_3, $H_{3'}$), 3.32 (dd, 2H, J_{gem} = 9,8 Hz, *J* = 5,1 Hz, H_{1a}, $H_{1'a}$), 3.26 (dd, 2H, J_{gem} = 9,8 Hz, *J* = 5,8 Hz, H_{1b}, $H_{1'b}$), 1,45 (q, 4H, J = 6,2 Hz, $H_{4/4'}$), 1,31 (sx, 4H, J = 7,2, Hz, $H_{5/5,}$), 0,87 (t, 6H, J = 7,3 Hz, $H_{6/6'}$) **^{13}C NMR** (100 MHz, [d_6]DMSO, 25 ºC): δ 72,3 (CH_2, $C_{1/1'}$), 70,2 (CH_2, $C_{3/3'}$), 68.6 (CH, C_2), 31,3 (CH_2, Hz, $C_{4/4'}$), 18,8 (CH_2, $C_{5/5'}$), 13,7 (CH_3, $C_{6/6'}$).

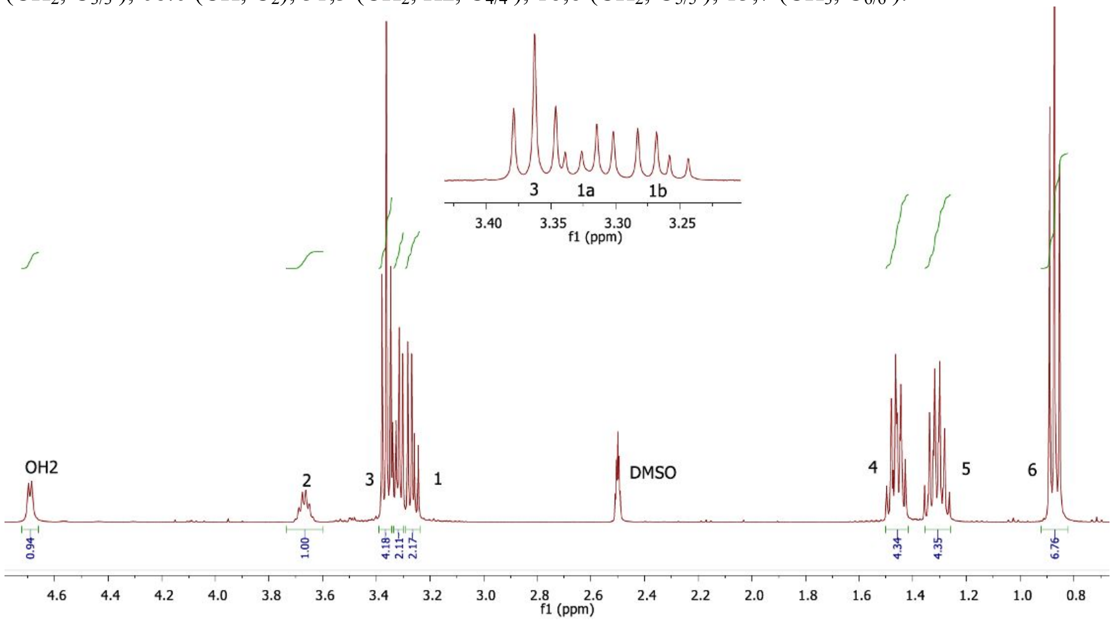

Figure S-23. ^{1}H NMR spectrum of 1,3-Dibutoxypropan-2-ol [**404**]

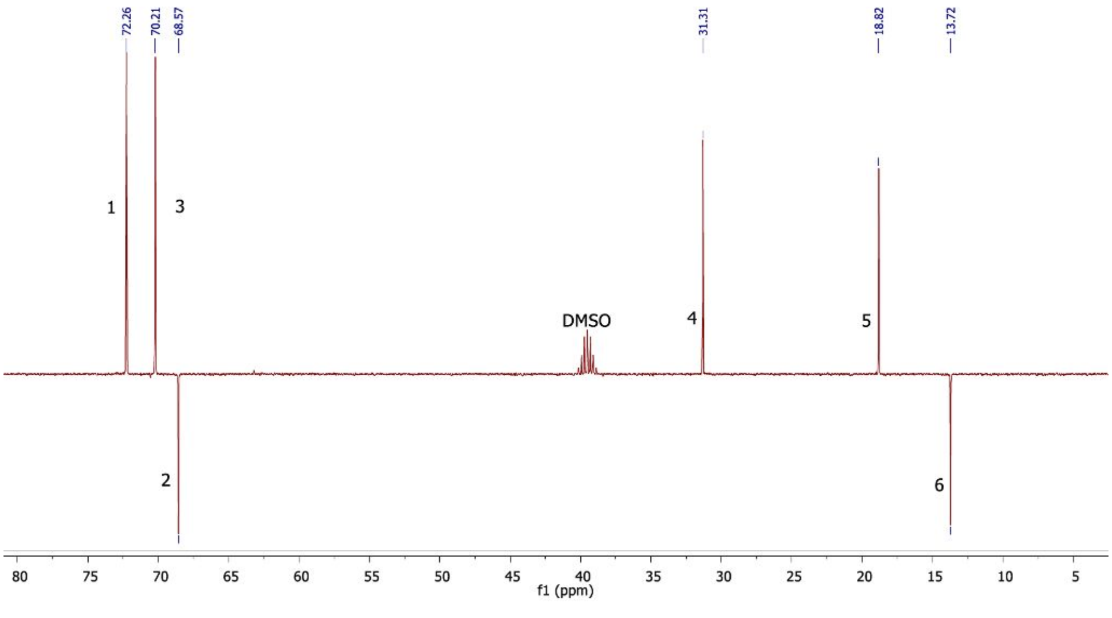

Figure S-24. ^{13}C NMR (APT) spectrum of 1,3-Dibutoxypropan-2-ol [**404**]

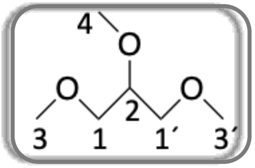

1,2,3-Trimethoxypropane [111], ^{1}**H NMR** (400 MHz, [d$_6$]DMSO, 25 ºC): δ 3,39-3,43 (m, 1H, H$_2$), 3,32-3,39 (m, 4H, H$_{1,}$ H$_{1'}$), 3,31 (s, 3H, H$_4$), 3,25 (s, 6H, H$_3$, H$_3$), ^{13}**C NMR** (100 MHz, [d$_6$]DMSO, 25 ºC): δ 78,5 (CH, C$_2$), 71,8 (CH$_2$, C$_{1/1'}$), 58,4 (OCH$_3$, C$_{3/3'}$), 57,0 (OCH$_3$, C$_4$),

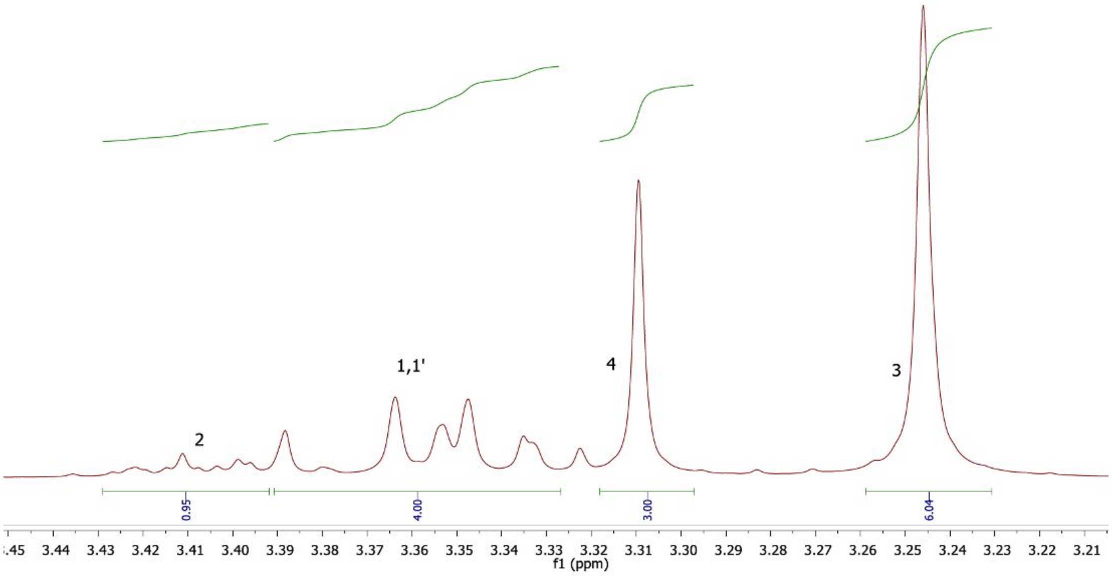

Figure S-25. ^{1}H NMR spectrum of 1,2,3-Trimethoxypropane [**111**]

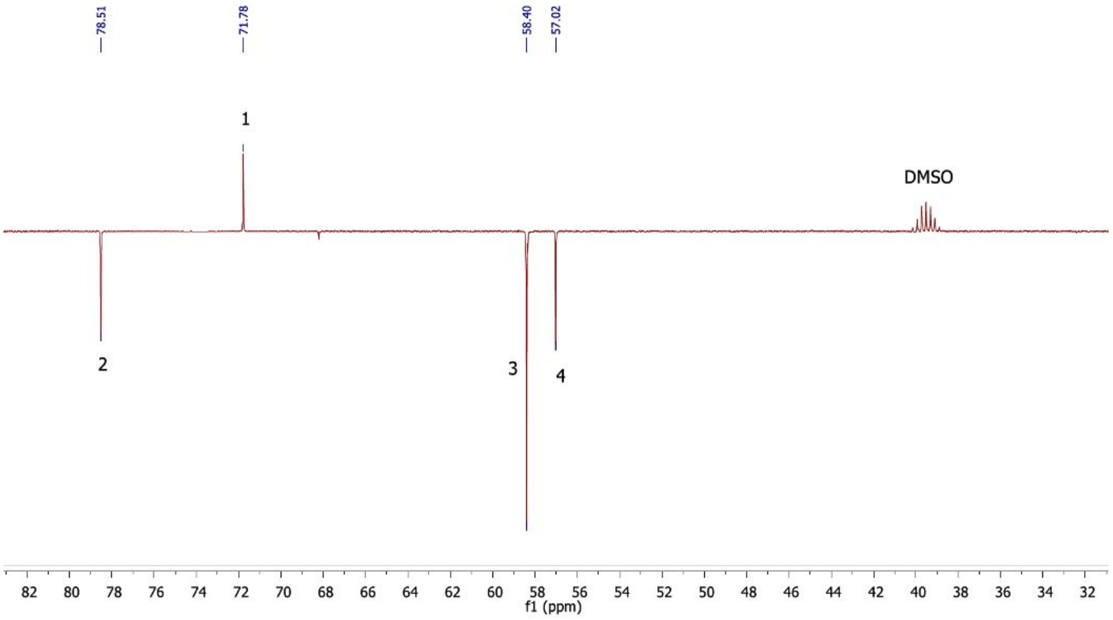

Figure S-26. ^{13}C NMR (APT) spectrum of 1,2,3-Trimethoxypropane [**111**]

3. HPLC calibrations of the studied acids

Area = 7.2672 x 10^9 · mg of coumaric acid. [r^2=0.9984] **Equation S1**

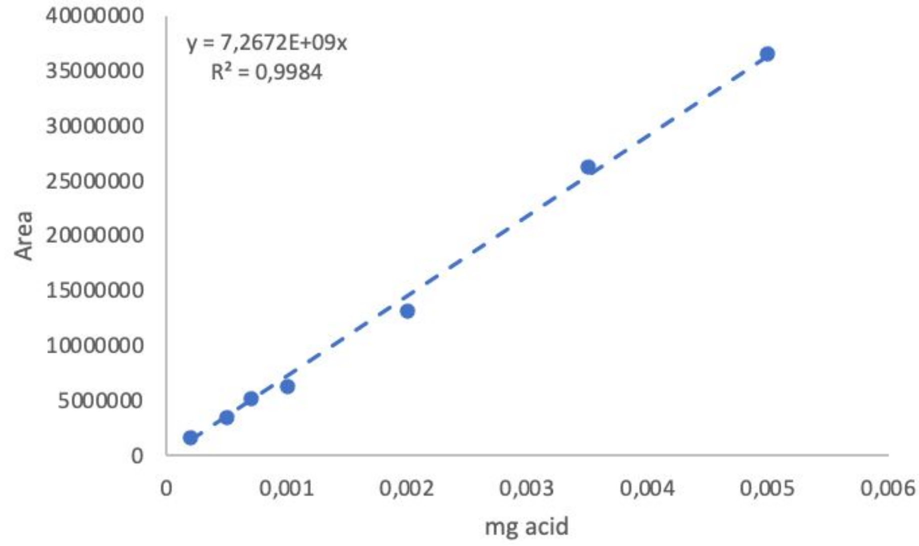

Figure S-27. Cumaric acid calibration curve

Area = 5.6049 x 10^9 · mg of ferulic acid [r^2 =0.9999] **Equation S2**

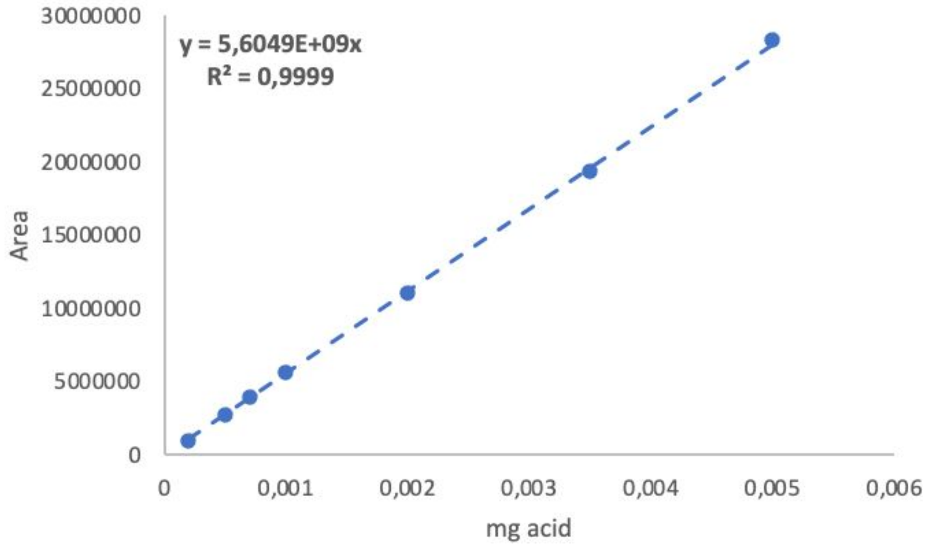

Figure S-28. Ferulic acid calibration curve

Area = 5.8166 x 10^9 · mg of caffeic acid. [r^2 = 0.9992] **Equation S3**

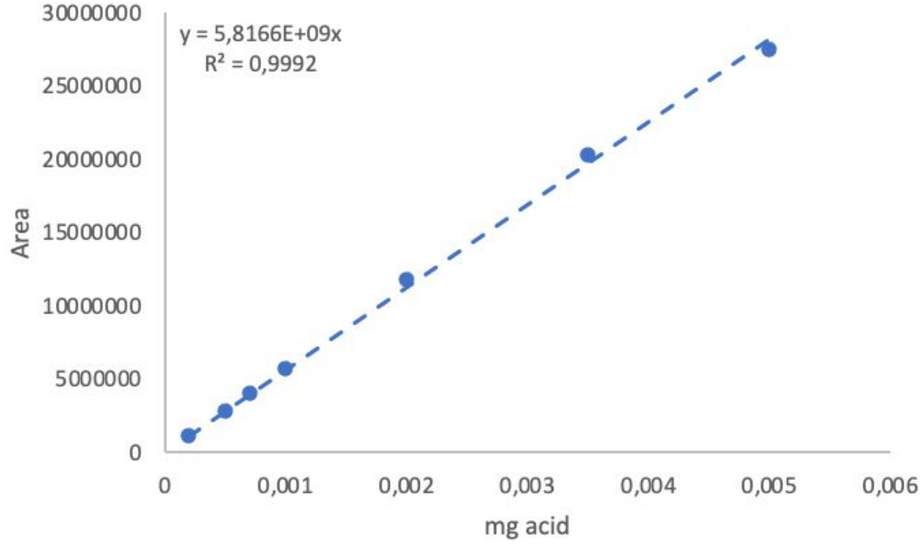

Figure S-29. Caffeic acid calibration curve

4. Solubility Values

Table S-1. Coumaric acid solubility in glycerol derived solvents

Solvent	Solubility (mg/mL)	%Std Dev
**100**	141.3	5.6
**200**	136.6	5.0
**3F00**	17.2	9.6
**300**	112.8	8.8
**3i00**	112.4	8.5
**400**	95.1	0.9
**101**	176.1	7.3
**202**	120.0	6.0
**3F03F**	2.7	0.3
**303**	74.6	7.6
**3i03i**	85.7	3.9
**404**	49.3	1.0
**111**	109.6	0.8
**EG**	103.0	1.7
**PG**	91.1	1.6
**EGMME**	255.9	5.7
**EGDME**	160.8	2.6

Table S-2. Ferulic acid solubility in glycerol derived solvents

Solvent	Solubility (mg/mL)	%Std Dev
**100**	103.4	4.2
**200**	88.6	6.3
**3F00**	18.6	1.5
**300**	64.4	6.3
**3i00**	74.2	2.9
**400**	56.8	4.8
**101**	127.7	8.9
**202**	65.5	8.3
**3F03F**	4.8	0.5
**303**	36.0	5.2
**3i03i**	0	3.0
**404**	25.7	1.7
**111**	95.4	4.1
**EG**	67.27	2.1
**PG**	37.66	0.9
**EGMME**	223.9	5.7
**EGDME**	142.55	6.5

Table S-3. Caffeic acid solubility in glycerol derived solvents

Solvent	Solubility (mg/mL)	%Std Dev
**100**	75.7	4.1
**200**	61.9	2.3
**3F00**	5.5	2.7
**300**	44.5	3.8
**3i00**	45.8	3.2
**400**	33.5	1.5
**101**	81.1	3.6
**202**	12.0	1.0
**3F03F**	0.3	0.1
**303**	17.2	0.9
**3i03i**	25.1	1.0
**404**	11.1	1.0
**111**	32.5	4.3
**EG**	42.0	2.1
**PG**	27.4	2.8
**EGMME**	98.9	3.2
**EGDME**	32.5	3.9

Table S-4. Coumaric acid solubility in aqueous solutions of **100**

x hydrotrope	Solubility (mg/mL)	%Std Dev
**0**	0.4	0.01
**0.1**	6.1	0.1
**0.2**	38.6	1.3
**0.3**	74.5	2.6
**0.4**	107.4	1.3
**0.5**	112.8	2.9
**0.6**	130.0	3.2
**0.7**	127.3	2.9
**0.8**	145.3	6.7
**0.9**	149.2	5.6

Table S-5. Ferulic acid solubility in aqueous solutions of **100**

x hydrotrope	Solubility (mg/mL)	%Std Dev
**0**	0.7	0.02
**0.1**	2.6	0.08
**0.2**	21.1	0.8
**0.3**	43.8	1.7
**0.4**	61.8	2.4
**0.5**	80.3	2,2
**0.6**	82.5	2,8
**0.7**	91.2	3.5
**0.8**	97.3	3.5
**0.9**	94.2	4.2

Table S-6. Caffeic acid solubility in aqueous solutions of **100**

x hydrotrope	Solubility (mg/mL)	%Std Dev
**0**	0.8	0.04
**0.1**	4.8	0.1
**0.2**	20.6	0.5
**0.3**	36.4	1.2
**0.4**	48.9	1.6
**0.5**	54.3	1.7
**0.6**	64.3	2.1
**0.7**	75.0	3.0
**0.8**	83.6	2.3
**0.9**	94.9	1.5

Table S-7. Coumaric acid solubility in aqueous solutions of **400**

x hydrotrope	Solubility (mg/mL)	%Std Dev
**0**	0.4	0.01
**0.1**	62.0	2.1
**0.2**	81.3	2.7
**0.3**	91.9	3.3
**0.4**	135.1	4.6
**0.5**	135.1	5.2
**0.6**	101.4	3.2
**0.7**	87.2	1.7
**0.8**	87.8	2.8
**0.9**	87.5	2.8

Table S-8. Ferulic acid solubility in aqueous solutions of **400**

x hydrotrope	Solubility (mg/mL)	%Std Dev
**0**	0.7	0.02
**0.1**	50.2	2.7
**0.2**	77.9	3.0
**0.3**	89.6	1.8
**0.4**	86.4	3.0
**0.5**	86.1	0.7
**0.6**	82.7	1.4
**0.7**	77.3	1.6
**0.8**	73.3	2.8
**0.9**	73.1	1.5

Table S-9. Caffeic acid solubility in aqueous solutions of **400**

x hydrotrope	Solubility (mg/mL)	%Std Dev
**0**	0.8	0.04
**0.1**	22.6	0.5
**0.2**	36.6	1.4
**0.3**	43.8	1.8
**0.4**	46.2	1.5
**0.5**	47.5	1.8
**0.6**	41.9	1.0
**0.7**	40.5	0.9
**0.8**	37.5	0.9
**0.9**	35.3	0.5

Table S-10. Coumaric acid solubility in aqueous solutions of **101**

x hydrotrope	Solubility (mg/mL)	%Std Dev
**0**	0.4	0.01
**0.1**	31.3	1.2
**0.2**	142.5	4.7
**0.3**	161.0	6.5
**0.4**	187.5	1.3
**0.5**	218.8	7.9
**0.6**	225.7	3.6
**0.7**	232.4	5.4
**0.8**	215.6	6.3
**0.9**	203.4	5.4

Table S-11. Ferulic acid solubility in aqueous solutions of **101**

x hydrotrope	Solubility (mg/mL)	%Std Dev
**0**	0.7	0.02
**0.1**	30.8	0.8
**0.2**	101.7	3.8
**0.3**	139.9	2.9
**0.4**	151.6	4.8
**0.5**	188.2	1.4
**0.6**	173.3	2.3
**0.7**	178.5	4.9
**0.8**	167.4	2.4
**0.9**	147.5	4.3

Table S-12. Caffeic acid solubility in aqueous solutions of **101**

x hydrotrope	Solubility (mg/mL)	%Std Dev
**0**	0.8	0.04
**0.1**	19.6	0.4
**0.2**	63.3	1.7
**0.3**	92.5	2.6
**0.4**	109.9	2.0
**0.5**	107.6	1.2
**0.6**	109.3	2.1
**0.7**	103.2	4.1
**0.8**	88.4	1.1
**0.9**	85.0	0.5

5. Cartesian coordinates for optimized geometries in Gaussian09

The coordinates for the most stable isomer of each of the ethers studied are shown below:

3-Metoxypropane-1,2-diol [100]

O	2.57953613948192	-0.27566038505064	-0.07080497499196
O	-0.86605694994366	-0.21515809678536	4.40087790908738
O	-2.43866077517912	0.86606573905991	-0.56939488752354
C	-0.71625592534046	-2.15909741969135	2.49657669166097
C	1.95898409406231	-2.44393811886701	1.44966957187071
C	-2.67763345392742	-1.66426914119989	0.42061828912672
C	5.02391052052420	-0.48965532138547	-1.21781524769422
H	-1.18656112196735	-3.95156633581719	3.44460855722761
H	2.06422432385019	-4.17989178919065	0.28825823676352
H	3.32305578248389	-2.62261905507694	3.02115578094522
H	-4.59215478460734	-1.80200827443686	1.20998879384277
H	-2.48221804415910	-3.08803761078385	-1.09200697599280
H	-1.12037067867044	1.36674775416626	3.47598155050775
H	5.11464922061723	-2.14426299962019	-2.48469358350481
H	6.50966182399734	-0.66483304171436	0.23521848644969
H	5.33511130622895	1.23971483977459	-2.31557047767887
H	-0.66605896185949	0.99909687474724	-1.11960691939725

3-Butoxypropane-1,2-diol [400]

O	-0.15816423506830	0.26160890775610	0.40721078268902
O	3.50513671672608	-2.10871810952877	4.00093739392688
O	2.03825001222887	3.00811283549729	4.17064824817747
C	-4.51366331836817	1.12429394671442	-0.69606165529510
C	4.10772113899607	-0.47075145361122	1.90795832028390
C	-1.99064844020720	0.08505020516727	-1.60035259421737
C	2.25185498275099	-0.78851620727520	-0.28168092239502
C	-5.74328606050598	-0.41029767196462	1.43530839508824
C	4.29196981397964	2.27472381937494	2.82021627496310
C	-8.30756729866114	0.65904985948904	2.25294481595500
H	-4.25753955057116	3.10524561717814	-0.09635927191327
H	-5.78753403531058	1.17190208331992	-2.34606971093024
H	5.97547038388296	-1.06892250729070	1.21162740062695
H	-2.18169620040838	-1.91877735052286	-2.16709297524365
H	-1.30004854407662	1.15685820457944	-3.25595769635353
H	2.04074726940576	-2.81504089516227	-0.74030168607348
H	3.00268876570937	0.19053239720408	-1.97082526216667
H	-5.97794841284898	-2.38247960909195	0.79859453207156
H	-4.45636416568775	-0.47230738108137	3.06984524449033
H	4.64333904617297	3.53787158760892	1.19711661405293
H	5.86514978840663	2.47205118984865	4.15950243920066
H	-9.65119895454659	0.68700893241038	0.66341792170140
H	-8.11818168323065	2.61074380773736	2.94973518173006

H	-9.15193165795757	-0.47661870246861	3.77573676689736
H	2.23247103568863	-1.20074774871915	4.98922868580646
H	0.64209504125524	2.64671069309303	2.99239713908837

3-(2,2,2-Trifluoroethoxy)propane-1,2-diol [3F00]

**F**	3.81149716745232	2.26107008193536	-1.53281734903816
F	6.82499674450853	-0.49614449804175	-0.88597316665525
F	4.56108928404975	0.88014887884781	2.31080700086822
O	2.44203545206419	-2.88787518733400	-3.01276052181322
O	-0.73856893537989	-5.80151953127767	-5.91851179732471
O	-4.32431230468622	-2.04809041231892	-7.34680084549793
C	0.11458475031597	-3.37887835174565	-6.83169835863866
C	0.64466253483583	-1.60121083864382	-4.63053217792587
C	-1.93486214998678	-2.37777489339473	-8.59588138125794
C	2.55710437098352	-1.99632938776016	-0.49264974816447
C	4.44316160566261	0.16580900141460	-0.16501282356521
H	1.86786227518035	-3.61491713143514	-7.94616520137638
H	-1.10111139462529	-1.21870225191632	-3.55934872227282
H	1.43204778319129	0.19215155673225	-5.33480756100154
H	-2.12129048178539	-3.69487915917684	-10.20291036558876
H	-1.38240102557377	-0.52073952440508	-9.34059673925146
H	3.21321512973151	-3.55924083725461	0.70524543507997
H	0.70522342454379	-1.34030651433462	0.19833066426786
H	0.31062909870087	-6.19518274008582	-4.44910171221866
H	-4.70466607010522	-3.65936109158338	-6.53099258231219

1,3-Dimethoxypropan-2-ol [101]

O	4.00793341822225	1.95373559539858	-1.31033385713573
O	-4.76137394748774	-0.12732663904439	-0.77189799650596
O	-0.59169901638311	3.17952223636362	0.72141530682531
C	-0.35525258713625	0.98528526215537	-0.87549757866283
C	2.32107675683332	-0.07719042231005	-0.70945098364982
C	-2.29059371067958	-0.96951784820920	-0.06956466455782
C	6.59892601900243	1.30064375982740	-0.87332747580724
C	-6.66402055954186	-1.89153051658658	-0.00595544255689
H	-0.73717552612017	1.52041906787425	-2.86103064656871
H	2.70807964507444	-0.77423617695896	1.22496328067463
H	2.56545119322954	-1.65774820049169	-2.05287499840063
H	-1.86428726064239	-2.79474965929554	-1.00089173914653
H	-2.18351631954605	-1.25449301360458	1.99991732947709
H	0.95068660597644	4.15865990290423	0.43557414707012
H	7.73299325152890	2.96968917942427	-1.34490970685017
H	6.91642738400161	0.78864127634204	1.12422668941441
H	7.18474438007938	-0.29496519806541	-2.08286796406943
H	-8.48896066281253	-1.12301383020306	-0.61819851596971
H	-6.68017326222117	-2.13532383485192	2.06734002670310
H	-6.36926580704666	-3.75668991517147	-0.89795813088336

1,2,3-Trimethoxypropane [111]

O	-0.79993853792373	2.68894631441178	-1.49621126492107
O	3.30494992167001	-1.13499170609451	-1.42921088151349
O	-4.52898253951941	-0.84134978460429	0.48445140975073
C	-0.32561275465230	0.89223758968652	0.48891924984095
C	2.46962145058873	0.21900363390061	0.75288541765202
C	-1.93887978367910	-1.42894329717631	-0.02787351425593
C	-0.20334763664911	5.23746240664870	-0.81127430732957
C	5.92161096399636	-1.79161754448231	-1.25016204399414
C	-6.14514902729079	-2.94194185944281	-0.04900511043362
H	-0.92893498045523	1.71355527427308	2.31778123152593
H	2.72277983313927	-0.94332075024382	2.47498230895989
H	3.60808470677760	1.95522559844911	1.00256311395910
H	-1.70993589829456	-2.02588334638181	-2.01655188179275
H	-1.30484904667357	-3.00068489516603	1.19687512183252
H	-1.27017820507087	5.82860379872604	0.88317537708243
H	1.83319731665313	5.50451258608580	-0.44892630475215
H	-0.75333630004316	6.43056262175769	-2.41519636046898
H	6.41697626356781	-2.80681845035991	-2.98846744276859
H	6.28233309302341	-3.02759440687847	0.39393484929064
H	7.11832675046651	-0.08883677193715	-1.08305824318874
H	-5.65065695943481	-4.59711773355988	1.12487556679314
H	-6.03555611369578	-3.49191896177242	-2.06039677905698
H	-8.08214457127362	-2.34909031394989	0.39182391969341

Coumaric Acid

O	-8.07225976473891	-0.59930208652637	0.67240748083818
O	8.62723514511110	-0.47456125613082	1.08371854825711
O	7.60948354912875	1.14235968385374	-2.76088668744831
C	-0.33610346749926	0.16807134101330	-0.80201202940822
C	-2.13593997445187	0.89633671364256	-2.63683873873023
C	-1.24704922978469	-0.81325786824196	1.51574595475100
C	-4.71958833335351	0.66252232917488	-2.20123347533231
C	-3.81610895141330	-1.05532673786686	1.97398020133761
C	-5.57034974615993	-0.31691704749403	0.11344402153782
C	2.33161968195888	0.45715370614713	-1.37213722779450
C	4.32260646853221	-0.14155943554775	0.12935647893023
C	6.92386493725712	0.26240847552063	-0.71741958861068
H	-1.48324567522235	1.65984615313303	-4.43979121954979
H	0.07781713070174	-1.39620375483649	2.98389991480081
H	-6.08125457424003	1.23544615725677	-3.64288488427041
H	-4.50526836237432	-1.81254065991188	3.76320659137554
H	2.76565645482347	1.25100566774599	-3.23477115749105
H	4.08202168936944	-0.94222520178026	2.01338215981861
H	-9.09786746506605	-0.02821183470009	-0.75261545927745
H	10.32473048742152	-0.15485536994838	0.40955927782568

Ferulic Acid

O	-4.78868378173423	-2.13192091859227	3.89333291131303
O	-7.90824142105767	0.48712623900353	0.88479277593256
O	8.69445623716601	-1.65474231240391	0.41811613381738
O	7.75847478171934	0.88647247133907	-2.91347175408215
C	-0.16570114674822	0.49238633936761	-0.71748152207483
C	-1.11401214775438	-0.87244005297425	1.38243778622064
C	-1.89110840118970	1.84094744332058	-2.24281198724570
C	-3.67960482801814	-0.87444665677139	1.91416390969063
C	-5.38627963813765	0.48988252234589	0.36169638627000
C	-4.47167776662341	1.84167261894200	-1.71148725456439
C	2.49653905877704	0.56359483696029	-1.36904826055599
C	4.43921209902113	-0.59551819513010	-0.16012226705518
C	7.03822629924757	-0.33498747925695	-1.06661513610154
H	0.15702694863273	-1.94012727386440	2.60884356359917
H	-1.18829979099968	2.90094978118563	-3.86625559130191
H	-5.80168788617274	2.88441911223693	-2.89162395460975
H	2.96951778179972	1.69227122872131	-3.03874885928146
H	4.15968938754925	-1.75917201844268	1.51810078370480
H	-3.51732541004814	-3.02493666330415	4.88658936109604
H	-8.19350473427669	-0.55069792432790	2.39217988800825
H	10.39279538245500	-1.40313713273510	-0.28239794016623

Caffeic Acid

O	-4.63352225363039	2.23682757659752	-2.48461419543447
O	-7.56988416314351	-1.06631393473742	-0.13613186721370
O	9.07683205384611	-0.27609915595694	-1.64936208514736
O	8.30126380766708	-3.77003510550650	0.72256586477715
C	0.25371014776363	-2.12670788969337	0.11751003262483
C	-3.38314936962911	0.31514046339495	-1.28622404522575
C	-0.78608964383904	-0.03596906387411	-1.20294445264131
C	-1.40263190722196	-3.82476723745268	1.33333273269021
C	-5.02264254364054	-1.41985855045493	-0.04890348601374
C	-4.01543391879940	-3.47735850998977	1.25337115987234
C	2.95116258418575	-2.59133685947380	0.26560141638050
C	4.84175455758558	-1.20305718248471	-0.77454887477268
C	-3.14519236657995	4.11021630797642	-3.79062712339466
C	7.48872435204134	-1.93039243598031	-0.45167584247751
H	0.45558589457225	1.30106769264267	-2.15558342865137
H	-0.62413450843890	-5.43833207762087	2.35497417525261
H	-5.29472035831558	-4.79145716010913	2.19432617593319
H	3.50336021417574	-4.26720610126119	1.34832875049756
H	4.48224669955840	0.49513986077582	-1.88625458945455
H	-2.03754354638214	3.23440921130980	-5.31634387253583
H	-1.88181223864038	5.09731065072595	-2.46711535036747
H	-4.50760712761194	5.44491035420441	-4.58890384049152
H	-7.89992837222654	0.46636632051519	-1.12698958813553
H	10.81185748213536	-0.86520279393944	-1.36528779027420

6. Comparison between experimental and COSMO-RS-calculated solubilities

Table S-13. Comparison between experimental and COSMO-RS calculated solubilities of coumaric acid

	Calculated solubility relative sreening (mg/mL)	Calculated solubility SLE (mg/mL)	Experimental solubility (mg/mL)
**100**	6.3	274.3	141.7
**101**	25.5	338.8	177.0
**111**	20.7	298.4	109.6
**3F00**	0.6	73.2	17.2
**400**	3.7	162.1	93.1

Table S-14. Comparison between experimental and COSMO-RS calculated solubilities of ferulic acid

	Calculated solubility relative sreening (mg/mL)	Calculated solubility SLE (mg/mL)	Experimental solubility (mg/mL)
**100**	1.5	344.6	103.4
**101**	5.5	431.9	127.7
**111**	5.4	398.5	95.4
**3F00**	0.2	125.8	4.8
**400**	1.1	229.2	56.8

Table S-15. Comparison between experimental and COSMO-RS calculated solubilities of caffeic acid

	Calculated solubility relative sreening (mg/mL)	Calculated solubility SLE (mg/mL)	Experimental solubility (mg/mL)
**100**	64.4	325.3	75.7
**101**	359.8	379.0	81.1
**111**	270.7	337.9	32.5
**3F00**	3.4	91.9	5.5
**400**	36.8	199.3	33.4

7. ^{1}H-NMR study of solute-solvent interactions in saturated solutions

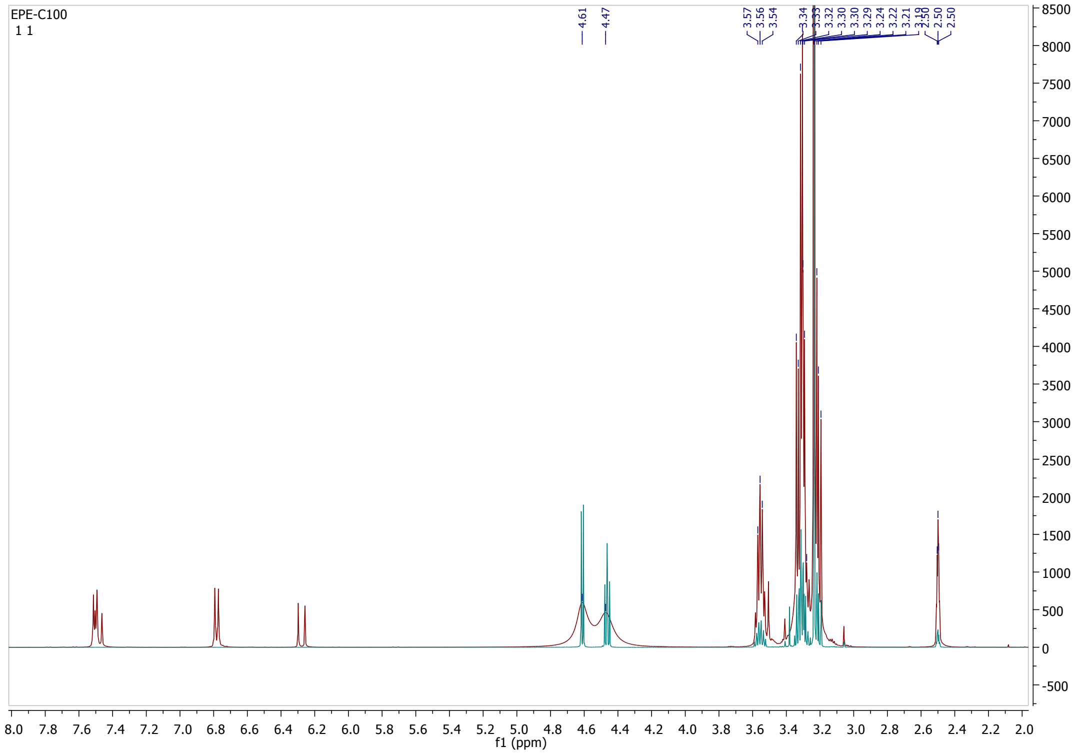

Figure S-30. ^{1}H-NMR of **100** monoether (green) and saturated solution of coumaric acid in **100** (red).

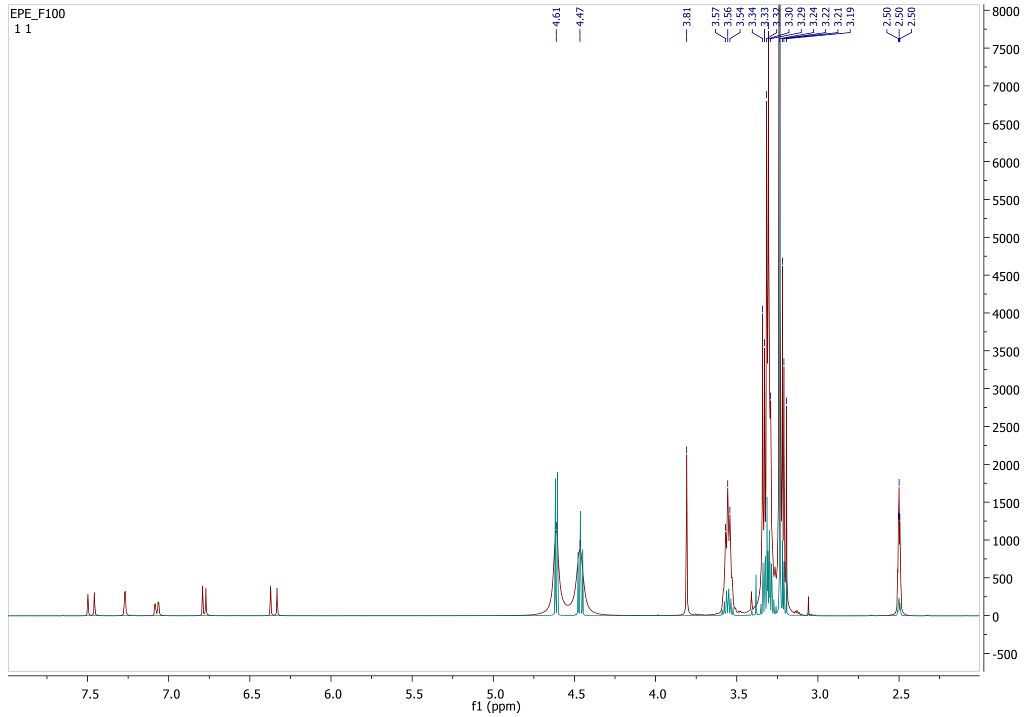

Figure S-31. ^{1}H-NMR of **100** monoether (green) and saturated solution of ferulic acid in **100** (red).

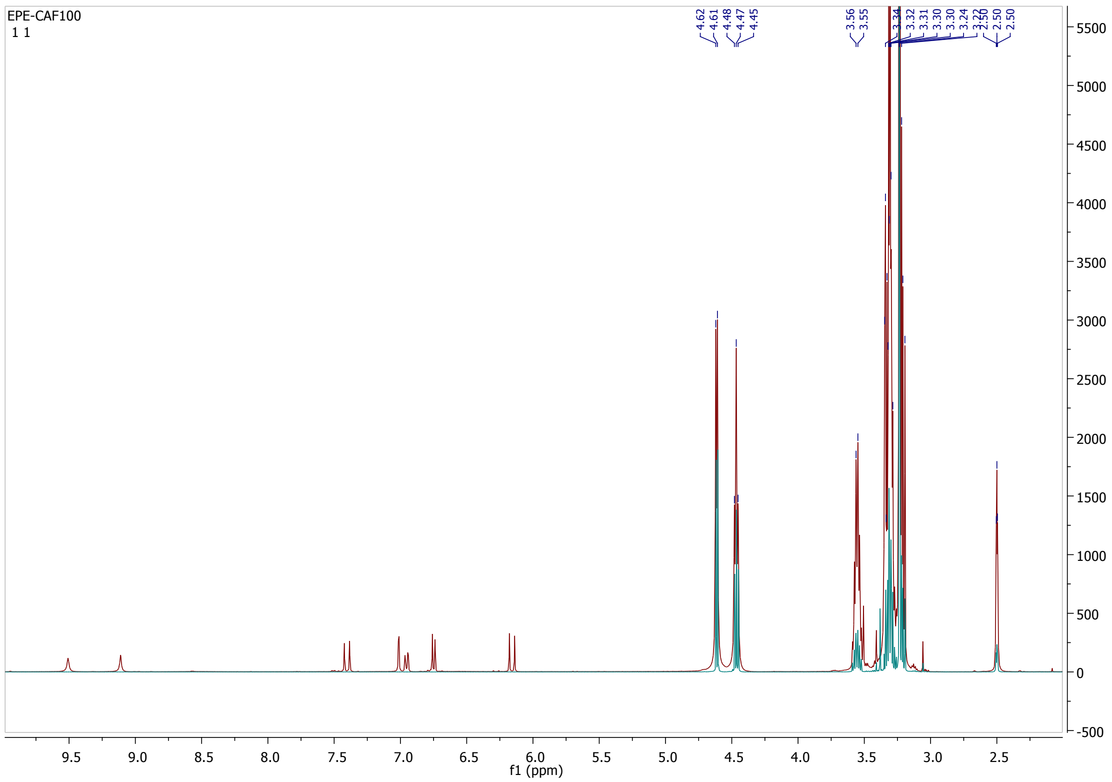

Figure S-32. ^{1}H-NMR of **100** monoether (green) and saturated solution of caffeic acid in **100** (red).

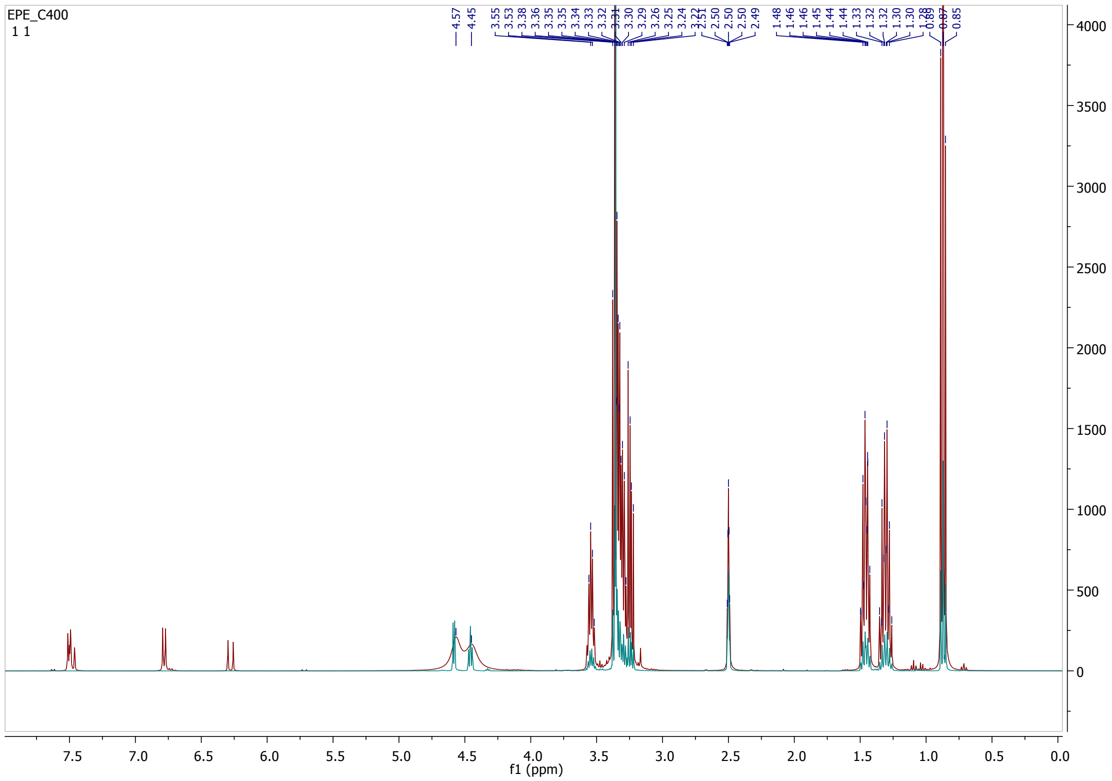

Figure S-33. ^{1}H-NMR of **400** monoether (green) and saturated solution of coumaric acid in **400** (red).

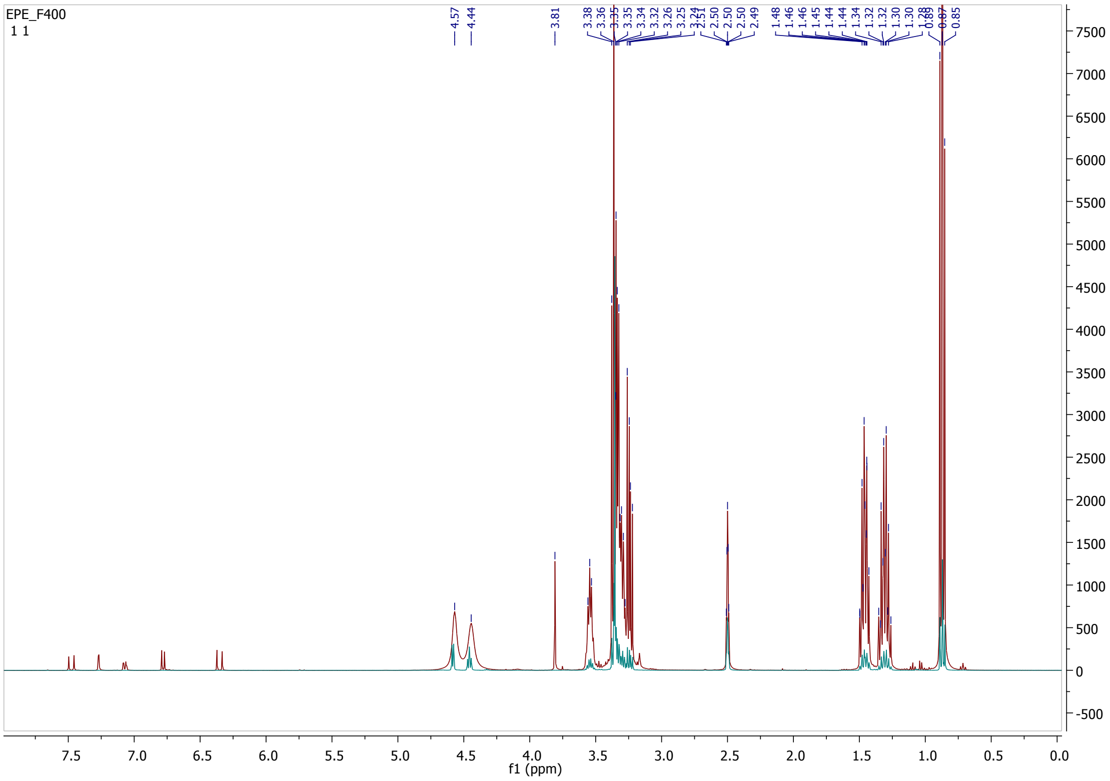

Figure S-34. ^{1}H-NMR of **400** monoether (green) and saturated solution of ferulic acid in **400** (red).

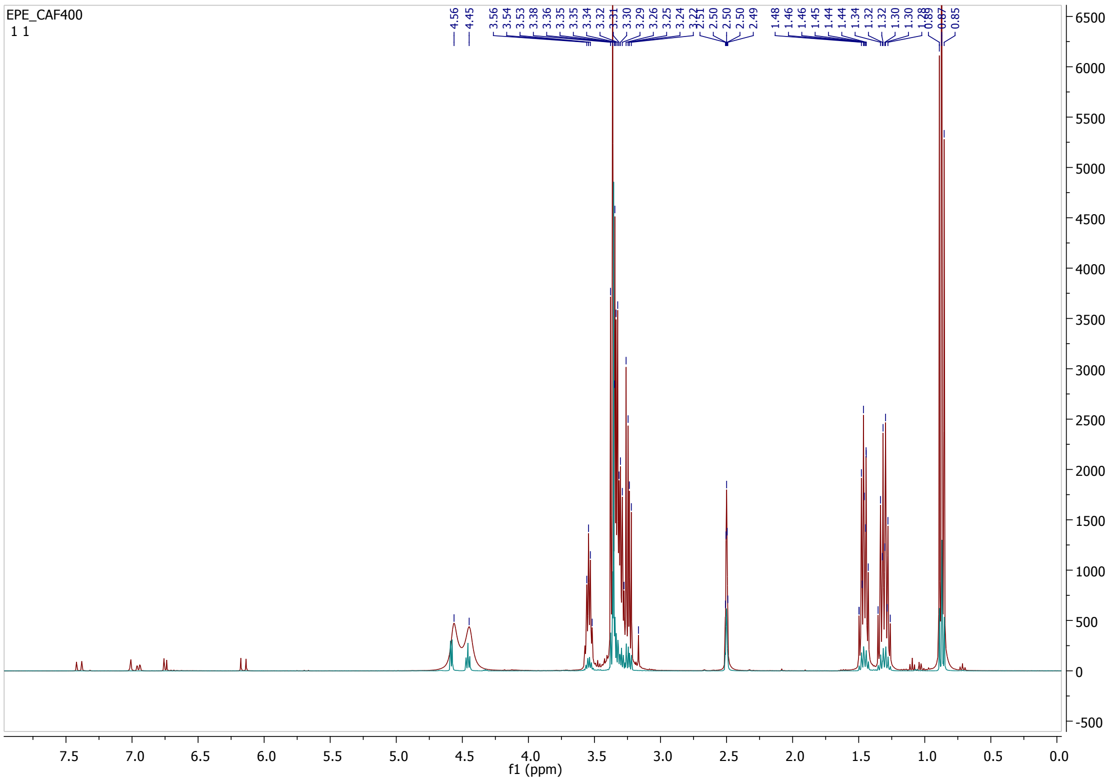

Figure S-35. ^{1}H-NMR of **400** monoether (green) and saturated solution of caffeic acid in **400** (red).
